# Burden of novel and ultra-rare missense variants in the NF-κB pathway genes associated to Ménière’s disease

**DOI:** 10.3389/fimmu.2026.1789753

**Published:** 2026-05-15

**Authors:** Pablo Cruz-Granados, Victoria Rivero de Jesus, Andres Soto-Varela, Rocio Gonzalez-Aguado, Jose A. Lopez-Escamez

**Affiliations:** 1 Meniere Disease Neuroscience Research Program, Faculty of Medicine & Health, School of Medical Sciences, The Kolling Institute, University of Sydney, Sydney, NSW, Australia; 2 Division de Otoneurologia, Department of Otorhinolaryngology, Consorci Sanitari Integral Moises Broggi Hospital, University of Barcelona, Barcelona, Spain; 3 Division of Neurotology, Department of Otorhinolaryngology, Complexo Hospitalario Universitario de Santiago de Compostela, Santiago de Compostela, Spain; 4 Department of Surgery and Medical-Surgical Specialities, School of Medicine, Universidade de Santiago de Compostela, Santiago de Compostela, Spain; 5 Health Research Institute of Santiago (IDIS), Santiago de Compostela, Spain; 6 ENT Department. Marqués de Valdecilla University Hospital, Santander, Cantabria, Spain; 7 IDIVAL, Cantabria, Spain; 8 Faculty of Medicine. University of Cantabria, Nedlands, Spain; 9 Otology & Neurotology Group CTS495, Division of Otolaryngology, Department of Surgery, Instituto de Investigación Biosanitaria, ibs.GRANADA, Universidad de Granada, Granada, Spain; 10 Sensorineural Pathology Programme, Centro de Investigación Biomédica en Red en Enfermedades Raras, CIBERER, Madrid, Spain; 11 Ear Science Institute Australia, Nedlands, WA, Australia

**Keywords:** autoimmune, Meniere disease, NF-κB, rare variant analysis, RNAseq

## Abstract

**Introduction:**

Ménière's disease (MD) is an inner ear disorder characterised by tinnitus, sensorineural hearing loss and dizziness, although around 60% of patients also exhibit and immune dysregulation. Transcriptomic studies have identified three distinct immune phenotypes in MD, classified based on systemic inflammation, cytokine profiles and association to autoinflammatory/autoimmune comorbidities. Furthermore, genetic studies have shown aggregation of rare variants in MD patients; however, the relationship between genetic factors and immune responses remains poorly understood.

**Methods:**

In this study, we analysed exome data pertaining to NF-κB pathway genes to identify ultrarare variants that could contribute to the immune phenotypes observed in MD. Furthermore, we retrieved MD epigenetic and transcriptomic data to search for the associated molecular signature. We performed protein modelling and receptor-ligand docking, as well as differential transcript usage (DTU) and splicing prediction.

**Results:**

We identified ultrarare heterozygous variants in *TLR9* (chr3:52222299 G>C and chr3:52223761 C>A), *TNFRSF1B* (chr1:12167150 C>T and chr1:12202100 G>A), and FAS (chr10:89003139 G>A and chr10:89008919 C>T) in MD patients. Splicing predictions suggested potential creation of cryptic donor/acceptor sites for *TLR9* and *TNFRSF1B* variants. Protein modelling predicted destabilising effects for all three proteins, with altered atomic interactions in *TNFRSF1B* and *FAS*. When docked, the *TLR9-TLR9* dimer and *FAS-FASGL* models, redistributed interfacial contacts, potentially reducing binding efficiency, whereas docked *TNFRSF1B-TRAF2* interactions were partially maintained.

**Conclusion:**

These findings suggest that MD distinct immune phenotypes based on systemic inflammation and may be linked to comorbid autoimmune/autoinflammatory disorders, with rare variants in *TLR9*, *TNFRSF1B* and *FAS* likely contributing to autoinflammation.

## Introduction

Ménière’s disease (MD) is an inner ear disorder characterised by episodic vertigo, tinnitus, and fluctuating sensorineural hearing loss (SNHL), which is particularly pronounced in the early stages of the disease (1). These symptoms can severely affect daily functioning, including work, social activities, and overall independence. Beyond its physical manifestations, MD has been strongly correlated with depression and anxiety, and the presence of these psychiatric symptoms contributes significantly to a reduction in quality of life (2).

MD is prevalent throughout Eurasia, though prevalence decreases toward the east. It affects 35 to 190 individuals per 100,000 globally (3). Several clinical subgroups have been described based on comorbidities—such as migraine and autoimmune conditions—and on the progression of SNHL (4, 5). Familial aggregation is found in ~6%–10% of cases (6, 7), with a burden of rare genetic variants in overlapping genes in ~29% of non-familial MD patients (8, 9). Most of these genes are involved in audiovestibular function and inner-ear homeostasis (10–12).

Three distinct immune phenotypes have been described in 60% of MD patients and are defined by systemic inflammation, cytokine profiles, and immune cell counts (13). (1) Autoinflammatory MD is characterised by elevated IL-1β levels and increased monocyte counts (14, 15). (2) Th2-driven MD shows high levels of Th2 cytokines (IL-4, IL-5, IL-13) and increased granulocyte counts (16–18). (3) MD is associated with comorbid immune-related diseases, such as ankylosing spondylitis or rheumatoid arthritis (4, 5, 19, 20).

Upregulated IL-1β in cultured HEI-OC1 cells promoted glutaminase (GLS) expression, resulting in increased glutamate release to the extracellular media (21). Furthermore, the study found that treating mice with an IL-1β blocker or GLS inhibitor reduced glutamate levels and ameliorated audiovestibular symptoms in mice with endolymphatic hydrops.

The nuclear factor kappa B (NF-κB) family of transcription factors is a key regulator of innate and adaptive immunity, with a pleiotropic effect on inflammation, cytokine production, and cell survival (22, 23). NF-κB dysregulation has been found to affect a number of autoimmune and inflammatory diseases, such as rheumatoid arthritis, systemic lupus erythematosus, Crohn’s disease, multiple sclerosis, and psoriasis. Several genes contribute to NF-κB pathway regulation, including *NFKB1*, *NFKB2*, *RELA*, *TNFRSF1A*, *TLR10*, *TNFSF12*, and *IKBKG*, and variants in these genes can influence both susceptibility and progression of immune-mediated disorders.

Several common variants in innate immune response genes may influence hearing outcome in MD. A *common* missense variant, chr4:38774486T>G (p.Ile369Leu), in the *TLR10* gene was found associated with MD, conferring a protective effect and reducing the risk of developing bilateral SNHL (24). Additionally, two regulatory variants in the *NFKB1* gene (chr4:102513096T>C and chr4:102554287G>T) showed a significant association with accelerated SNHL progression in patients with unilateral MD (25).

In a case–control study, a common noncoding variant chr6:31058178T<C (rs4947296), was significantly associated with bilateral MD (26). This signal is a trans-expression quantitative trait locus (eQTL) in peripheral blood mononuclear cells (PBMCs), inducing upregulation of 31 differentially expressed genes from the NF-κB non-canonical TWEAK/Fn14 pathway. *In-vitro* experiments suggest that the risk genotype (CC) upregulates *NFKB1* and *TNFRSF12A* compared to the protective genotype. A 2025 study using single-cell RNA sequencing of the stria vascularis (SV) found that TWEAK is released by intermediate cells, whereas marginal and spindle cells express its receptor *TNFRSF12A* (Fn14) (27).

Moreover, new evidence suggests that genes in the NF-κB pathway may contribute to the development of inner ear disorders. In particular, higher *NFKB2* expression has been associated with sudden SNHL in a case-control study (28).

Furthermore, genes involved in immune responses have been identified in the SV and linked to MD (8, 27, 29), with some harbouring rare variants that may play a critical role in the altered immune response observed in certain patients (30). In this study, we focused on rare variants in genes associated with the NF-κB pathway, which may contribute to the immune dysregulation present in MD.

## Materials and methods

### Human ethics

The Human Ethics Research Committee (2023/HE000199) from the University of Sydney approved the protocol for this study on 21 June 2023. Informed consent was obtained from all participants to donate blood samples and perform genetic analyses. This work was performed under the standards of the Declaration of Helsinki.

### Dataset retrieval

Whole-genome and whole-exome sequencing datasets from both sporadic and familial MD cases were retrieved from previously published studies. Each dataset was comprised of MD patients from distinct populations, including 891 Non-Finnish European (NFE) (511 American cohort and 380 Spanish cohort) (8, 11), 16 East Asian (EAS) (31), and 22 Admixed American (AMR) (32). All patients were diagnosed according to the 2015 MD diagnostic criteria from the Bárány Society International Classification Committee for Vestibular Disorders (1) (**Tables 1**, **2**).

**Table 1:** Published datasets used in this study with sample size, mean age (SD), sex distribution, and data quality controls.

Study	Population	Sample size	Mean age (SD)	Sex			Data quality controls			
				Male	Females	Missing	Allele balance	Genotype quality	Depth/coverage	Variant quality score
8	Non-Finnish European (Spanish)	380	39.4 (13.5)	168 (44.1%)	194 (50.9%)	18 (4.7%)	0.2–0.8 for heterozygous genotypes	≥ 20	≥ 10 (≥ 5 for haploid genotypes)	first tranche (90% truth sensitivity)
31	East Asian	16	39.9 (13.6)	6 (40%)	10 (60%)	–				
32	Admixed American	22	39.5 (11.9)	8 (36.4%)	14 (63.6%)	–				
11	Non-Finnish European (American)	511	NA	NA	NA	NA	NA	NA	NA	NA

*SD, standard deviation.

**Table 2:** Diagnostic criteria for MD according to the Bárány Society. Adaptad from Lopez-Escamez et al. (1).

Definite MD
A. Two or more spontaneous episodes of vertigo, each lasting 20 min to 12h
B. Audiometrically documented low- to medium-frequency sensorineural hearing loss in one ear, defining the affected ear on at least one occasion before, during or after one of the episodes of vertigo
C. Fluctuating aural symptoms (hearing, tinnitus, or fullness) in the affected ear
D. Not better accounted for by another vestibular diagnosis
Probable MD
A. Two or more episodes of vertigo or dizziness, each lasting 20 min to 24h
B. Fluctuating aural symptoms (hearing, tinnitus or fullness) in the affected ear
C. Not better accounted for by another vestibular diagnosis

### Variant burden in the NF-κB pathway

A curated set of 63 genes encompassing both core components of the canonical and non-canonical NF-κB pathways, as well as key upstream regulators and modulators, was selected for gene burden analyses (GBA) to identify an excess of rare missense and loss-of-function (LoF) variants (stop-gained, frameshift, splice-site, or start-lost) (**Supplementary Table 1**). Allele counts and allelic frequency of variants from the genome data of gnomAD v4.1 for NFE and global populations were retrieved to be used as controls for the GBA performed in the NFE (Spanish cohort). For genes showing significant burden in MD cases, variants were further filtered based on population-specific allele frequencies from gnomAD and the Collaborative Spanish Variant Server (CSVS) (33). Selected variants were then examined in the NFE (American cohort), EAS, and AMR cohorts for replication. We analysed co-occurring variants within the same genes across individuals to determine potential haplotype formation.

Gene-level burden was assessed by comparing aggregated allele frequencies between cases and controls using Fisher’s exact test, with statistical significance determined after Bonferroni correction and requiring an adjusted *p*-value > 0.05 and an odds ratio (OR) < 1, as described by (31).

A GBA was performed on synonymous variants to determine whether any of these variants could be part of a haplotype shared among several individuals. Novel variants were validated using individual BAM files with the IGV browser (**Supplementary Figures 1**–**14**).

### Epigenetic modifications in the NF-κB pathway genes

We retrieved whole-genome bisulfite sequencing (WGBS) data to identify methylated cytosines and assess methylation patterns within CpG islands (34). In addition, we analysed pseudo-bulk ATAC-seq data to identify upregulated chromatin-accessibility peaks associated with the MD autoinflammatory phenotype (15) in order to assess whether the selected genes may contribute to the autoinflammatory mechanisms underlying MD.

### Bulk RNA expression of selected NF-κB pathway genes

Bulk RNA-seq FASTQ files from MD samples carrying the selected variants (35) were quantified with Salmon v1.10.1 (36) and imported into R using tximport v1.34.0 (37) for transcript-level analysis. Differential transcript usage (DTU) was assessed using the rnaseqDTU v1.26.0 (38) and DRIMSeq 1.34.0 framework (39), and selected transcript identifiers were obtained from Ensembl (https://www.ensembl.org/index.html) via biomaRt v 2.62.1 (40). Normalised transcript counts generated with DESeq2 were extracted and filtered for genes in our curated set.

### NF-κB pathway gene co-expression partners

Single-cell RNA-seq FASTQ files from MD samples and controls (15) were quantified following the methodology previously described. Using normalised DESeq2 data, we performed weighted gene co-expression analysis with the WGCNA package in R, focusing specifically on the curated set of NF-κB pathway genes, plus cytokines that define the autoinflammatory and Th2 MD phenotypes (41). To further uncover predictive relationships, we employed elastic-net regularised logistic regression through the glmnet package, partitioning the data into training and test sets and assessing model performance using receiver operating characteristic curves. By integrating the insights from these complementary approaches, we identified biologically relevant genes within the NF-κB pathway, shedding light on potential key regulators of the underlying molecular processes.

### Inner ear expression of NF-κB pathway genes

The gEAR portal (42) was used to retrieve gene expression data from the SV dataset (43) and inner ear organoid dataset (44) to assess NF-κB pathway gene expression in the inner ear. A filter of ±1 logfold change (log_2_ FC) was used to identify significant expression changes. Additionally, single-cell RNA sequencing of SV marginal, basal, and intermediate cells in P30 mice (27) was used to further evaluate the gene expression at the cellular level.

### Pathogenicity assessment

To predict splice sites in NF-κB pathway genes, the SpliceAI Lookup Browser (https://spliceailookup.broadinstitute.org/#) was employed, incorporating predictions from *Pangolin* (45) and *SpliceAI* (46). A 500-base pair (bp) window up and downstream from the variant was applied. To validate splicing, the *Human Splice Finder Pro* (47) was used. Predictions considered *cis*-acting regulatory elements, including Intronic Splicing Enhancers (ISEs), Intronic Splicing Silencers (ISSs), Exonic Splicing Enhancers (ESEs), Exonic Splicing Silencers (ESSs), and branch points associated with the variants of interest.

gnomAD v2.1 (48) was used to retrieve exome data from diverse populations [NFE, EAS, AMR, Finnish European (FIN), Southeast Asian (SAS), Ashkenazi Jews (ASH), and Sub-Saharan African (AFR)]. To evaluate protein conservation at the selected mutations, constrained regions with an accumulation of missense variants in the genes of interest were identified by calculating the density of variants across the coding sequence (CDS), following the methodology described by 49.

### Protein modelling

Wild-type toll-like receptor 9 (*TLR9*), tumour necrosis factor receptor superfamily member 1B (*TNFRSF1B*), and tumour necrosis factor receptor superfamily member 6 (*FAS*) computational models were retrieved from Alphafold2 (50). The quality of the structure was assessed using the SAVES server v6.1 (https://saves.mbi.ucla.edu/), ERRAT, WHATCHECK, and PROCHECK. Mutant protein models were generated by homology using MODELLER v10.6 (51), and the protein sequence was extracted from the Uniprot server (52). The best model was chosen according to MODELLER in-built quality check DOPE and GA341 scores. Protein models were visualised using *PyMOL* Open Source (53).

Dynamut2 (54) was used to assess the predicted stability change (ΔΔG^Stability^) between wild-type and mutant protein models.

### Molecular docking

For toll-like receptor 9 and tumour necrosis factor receptor superfamily member 6 models of the extracellular domains were generated using homology modelling in MODELLER v10.6 based on their respective sequences. Conversely, a model of the intracellular domain of tumour necrosis factor receptor superfamily member 1B was also generated. Additionally, models of the extracellular and intracellular domains of their respective ligands were generated. The first and last amino acids of each domain were determined from previously reported literature (55–64).

Wild-type and mutant PDB files from the selected proteins were uploaded into the ClusPro server (65) for molecular docking. Ligand wild-type PDB files were retrieved from the AlphaFold server. *PRODIGY* v2.4 (66, 67) was used to predict the binding affinity in protein-protein interactions. Arpeggio was used to determine ligand-receptor and receptor-receptor atomic interactions (68).

### Homology across species

A variant-centred human-mouse homology analysis was performed to investigate whether species-specific differences in variant conservation may influence the variability in the immune dysregulation caused by the NF-κB pathway genes, according to the methodology as described by (69). We adjusted the methodology to compare *Homo sapiens* to *Pan troglodytes* subspp. (chimpanzee).

## Results

**Figure 1:**
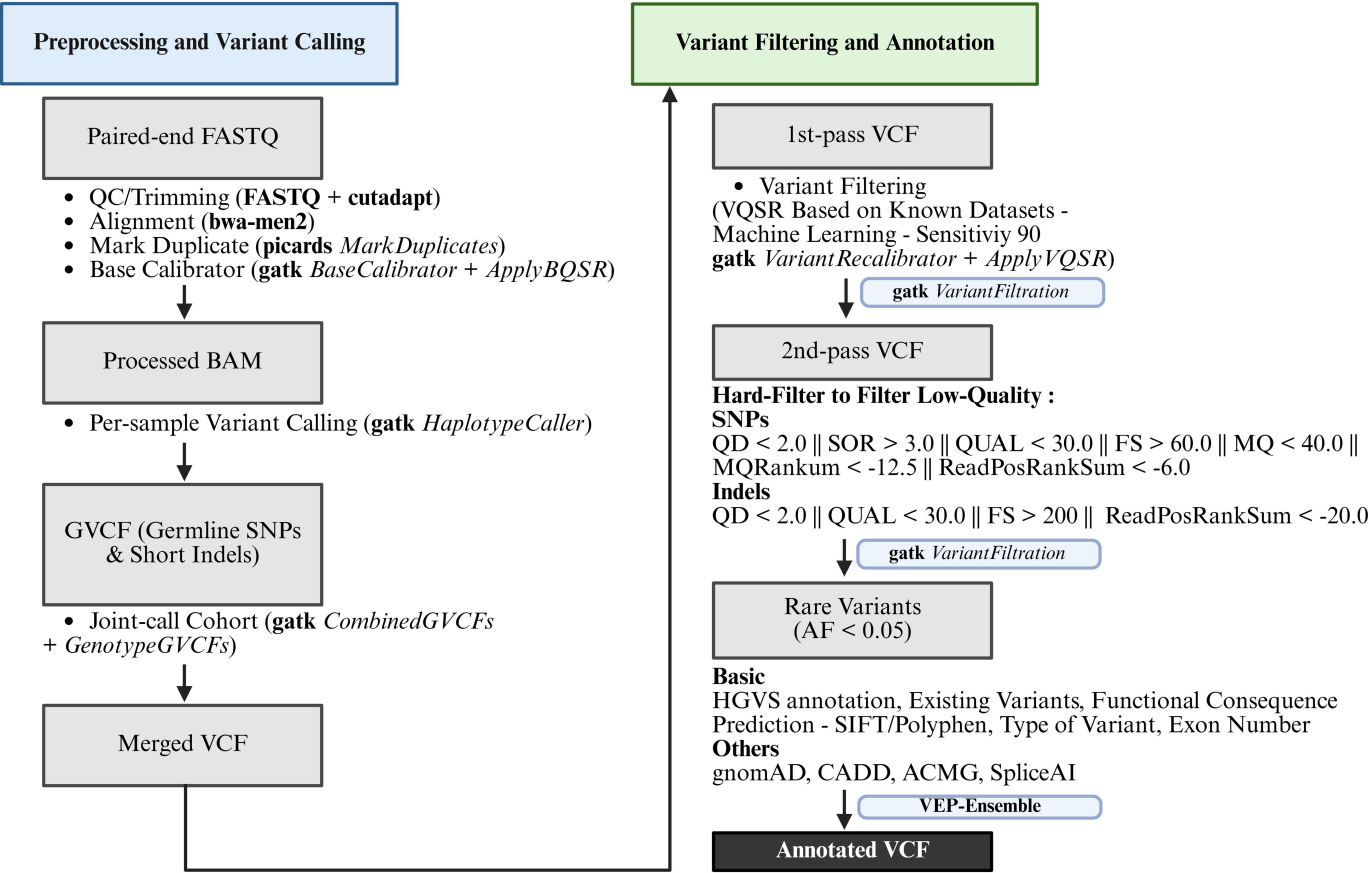
Comprehensive workflow for variant calling, filtering, and annotation from FASTQ to annotated VCF adapted from Pham et al 2026 (31). FASTQ, FAST quality; VCF, variant call format.

We identified 12 of 63 (19%) NF-κB pathway genes (*FAS*, *IKBKE*, *LTBR*, *TLR1*, *TLR2*, *TLR4*, *TLR5*, *TLR9*, *TNFAIP3*, *TNFRSF1B*, *TNFSF12*, and *TRAF2*) that exhibited a significant burden of novel and ultra-rare missense variants in the Spanish cohort (*N* = 371). Variants were filtered using the workflow shown in **Figure 1**: the initial variant call set underwent quality control to remove low-confidence calls, followed by restriction to candidate NF-κB genes and exclusion of variants with allele frequency >0.05 in CSVS. Remaining variants were prioritised based on the (OR) >5 and false discovery rate (FDR) < 0.05 compared to gnomAD NFE and CSVS populations. Across these genes, we detected 152 ultra-rare variants, including five nonsense variants and 147 missense substitutions (**Table 3**).

**Table 3:** Gene burden analysis showing enrichment missense and LoF variants in the NF-κB pathway-related genes.

Symbol	N° variants	AC	MAF	NFE			Global			CSVS		
				MAF	OR (95% CI)	FDR	MAF	OR (95% CI)	FDR	MAF	OR (95% CI)	FDR
*FAS*	2	7	0.009	3.68E-04	25.917 (9.875 to 57.679)	1.49E-06	0.022	0.419 (0.168 to 0.868)	0.883	0.002	5.957 (2.006 to 16.121)	0.043
*IKBKE*	6	6	0.008	2.57E-05	315.957 (87.863 to 1071.929)	3.54E-11	3.12E-05	261.224 (85.132 to 690.799)	2.93E-11	2.52E-04	32.230 (3.905 to 1473.250)	0.005
*LTBR*	4	6	0.008	1.69E-04	48.232 (16.003 to 121.927)	5.65E-07	0.007	1.181 (0.432 to 2.588)	1	5.05E-04	16.139 (4.601 to 53.137)	9.17E-04
*TLR1*	5	6	0.008	2.18E-04	37.458 (13.274 to 85.651)	1.45E-06	1.27E-04	64.023 (22.834 to 145.086)	5.86E-08	5.73E-04	14.217 (3.937 to 49.568)	0.002
*TLR2*	6	6	0.008	3.75E-04	21.738 (7.770 to 49.189)	3.18E-05	8.65E-04	9.412 (3.427 to 20.721)	0.003	4.91E-04	16.588 (3.923 to 80.043)	0.003
*TLR4*	7	9	0.012	1.23E-04	99.166 (42.392 to 209.281)	1.55E-13	9.99E-05	122.923 (53.846 to 247.711)	1.54E-14	4.09E-04	29.976 (8.995 to 114.222)	5.15E-07
*TLR5*	5	5	0.007	0	Inf (168.889 to Inf)	2.46E-10	4.93E-05	137.617 (39.011 to 397.858)	6.69E-08	4.30E-04	15.784 (3.939 to 57.899)	0.005
*TLR9*	7	8	0.011	7.84E-05	139.046 (55.085 to 307.687)	4.39E-13	0.003	4.011 (1.723 to 7.960)	0.059	6.61E-04	16.472 (5.373 to 50.563)	5.37E-05
*TNFAIP3*	4	7	0.009	2.09E-04	45.434 (17.435 to 100.090)	3.08E-08	0.002	5.691 (2.274 to 11.831)	0.016	6.80E-04	13.990 (4.586 to 39.676)	3.34E-04
*TNFRSF1B*	8	8	0.011	3.92E-05	278.400 (102.934 to 699.754)	4.31E-15	2.13E-04	51.095 (21.659 to 103.116)	5.19E-10	6.55E-04	16.629 (5.424 to 51.058)	5.02E-05
*TNFSF12*	2	5	0.007	2.21E-04	30.695 (8.709 to 89.219)	1.04E-04	4.02E-04	16.883 (5.281 to 41.769)	9.92E-04	2.56E-04	26.449 (2.954 to 1243.725)	0.028
*TRAF2*	9	9	0.012	1.88E-04	65.240 (28.423 to 132.353)	4.75E-12	1.83E-04	67.233 (29.976 to 131.993)	2.58E-12	6.80E-04	18.039 (6.158 to 53.847)	7.24E-06

LoF, loss-of-function; AC, allelic count; MAF, maximum allelic frequency; NFE, non-Finnish European; CSVS, collaborative Spanish variant server; OR (95% CI), odds ratio (95% confidence interval); FDR, false discovery rate.

We selected and ranked 40 missense variants in *FAS*, *IKBKE*, *LTBR*, *TLR1*, *TLR2*, *TLR4*, *TLR5*, *TLR9*, *TNFRSF1B*, *TNFAIP3*, *TNFSF12*, and *TRAF2* and two nonsense variants, one in *TLR2* and one in *TLR4*, which met both statistical and biological significance criteria relative to the gnomAD NFE population (**Supplementary Table 2**). Variants detected in *IKBKE*, *TLR2*, *TLR4*, *TLR5*, *TLR9*, *TNFRSF1B*, *TNFSF12*, and *TRAF2* were novel, absent from the gnomAD NFE and global datasets as well as from the CSVS database. Four variants identified in *IKBKE*, *TLR5*, and *LTBR* were absent from gnomAD but present at low frequency in the Spanish general population.

When we compared to the NFE and the general Spanish population, we found a burden of rare synonymous variants in the *LTBR*, *TLR2*, *TLR5*, and *TLR9* genes. Furthermore, we found 11 significant synonymous variants in *FAS*, *IKBKE*, *TLR1*, *TLR5*, *TLR9*, *TNSF12*, and *TRAF2* (**Supplementary Tables 3**, **4**).

Two individuals harboured one ultrarare heterozygous missense variant each (Patients 1 and 2). In addition, we identified a second variant in the same gene in the same individual (**Supplementary Table 5**). Patient 1 carried two variants in *TLR9* (NC_000003.12 g.52222299G>C, p.Leu673Val and NC_000003.12 g.52223761C>A, p.Arg185Ser), both located in exon 2. Patient 2 carried two variants in *TNFRSF1B* (NC_000001.11 g.12167150C>T, p.Ala20Val and NC_000001.11 g.12202100G>A, p.Arg345Gln), located in the first and last exons, respectively.

When looking upon the ultra-rare synonymous variants, we found two more individuals that have a synonymous variant (NC_0000010.11 g.89003139G>A) and a missense variant (NC_0000010.11 g.89008919C>T, p.Ile122Thr) in *FAS* (**Table 4**).

**Table 4:** Burden of variants in the *FAS* gene.

Symbol		*FAS*	
Position		89003139	89008919
Ref		G	C
Alt		A	T
Consequence		synonymous	missense
Amino Acid Change		–	p.Ile122Thr
AC		2	2
MAF		0.003	0.003
CADD		0.503	13.49
ACMG		Bening	Bening
NFE	MAF	2.65E-04	1.62E-04
	OR (95% CI)	10.209 (1.147 to 42.740)	16.709 (1.796 to 76.854)
	FDR	0.039	0.017
Global	MAF	0.02	0.008
	OR (95% CI)	0.132 (0.016 to 0.480)	0.352 (0.042 to 1.278)
	FDR	1.91E-04	0.278
CSVS	MAF	4.91E-04	4.91E-04
	OR (95% CI)	5.500 (0.398 to 75.851)	5.500 (0.398 to 75.851)
	FDR	0.23	0.23

AC, allelic count; MAF, maximum allelic frequency; NFE, non-Finnish European; CSVS, collaborative Spanish variant server; OR (95% CI), odds ratio (95% confidence interval); FDR, false discovery rate.

None of the missense variants were observed in the EAS or AMR cohorts; however, two individuals in the AMR cohort carried the synonymous variant chr10:89003139 G>A in *FAS*.

### Methylation in MD individuals in selected genes

We found no methylated CpGs in MD cases in selected variants in a 5000-base-pair window. However, in the pseudo-bulk ATAC-seq data, we found one chromatin-accessibility peak upregulated in the *TNFRSF1B* gap chr1-12183860–12184881 region (log_2_FC = 1.76; FDR = 2.56E^−10^) (**Supplementary Table 6**). We did not find any upregulated or downregulated ATACseq regions in *TLR9* and *FAS*.

### Clinical phenotypes

Patient 1 was a female in her 6th decade of life. The age of onset of the vertigo episodes was in their mid-20s. The individual reported tinnitus in the right ear, bilateral synchronic SNHL with a pure tone average (PTA) of 108 decibel hearing level (dB HL) in the right ear and approximately 73 dB HL in the left ear (**Supplementary Figure 15**). Comorbidities reported were migraine without aura, rheumatoid arthritis, and Raynaud syndrome. Furthermore, she has a history of allergies to pollen, dust and dust mites, and seafood. The individual reported a maternal history of episodic vertigo and migraine, without SNHL. This patient also reported Tumarkin’s otolithic crisis. This patient was classified as bilateral clinical subgroup 5.

Patient 2 was a male in their sixth decade of life. The age of onset was in their late 20s. The individual reported permanent tinnitus in both ears, bilateral synchronic SNHL with PTAs of ≈78 dB HL and ≈55 dB HL in the right and left ear, respectively (**Supplementary Figure 16**), vertigo, no headache, nor associated autoimmune disorders not Tumarkin’s. This patient was classified as bilateral clinical subgroup 2.

Patient 3 was a male in their late 40s with a reported age of onset on their late 30s. The individual reported non-permanent tinnitus, vertigo, and unilateral hearing loss and a more severe PTA in the right ear (≈62 dB HL), compared to the left ear (≈14 dB HL) (**Supplementary Figure 17**). Only the low frequencies were affected, which is characteristic of the early stages of the disease. The individual reported a history of migraine with headache and Tumarkin’s, and no associated autoimmune disorders. This patient was classified as unilateral clinical subgroup 4.

Patient 4 was a male in their sixth decade of life, diagnosed in their mid-50s. The individual presented episodes of vertigo with bilateral permanent tinnitus and hearing loss, and PTAs were relativity similar in the right ear (50 dB HL) and left ear (43 dB HL) (**Supplementary Figure 18**). The individual did not report a history of headache, autoimmune comorbidities, or Tumarkin’s episodes. This patient was considered as bilateral subgroup 1.

### Differential transcript usage

**Figure 2:**
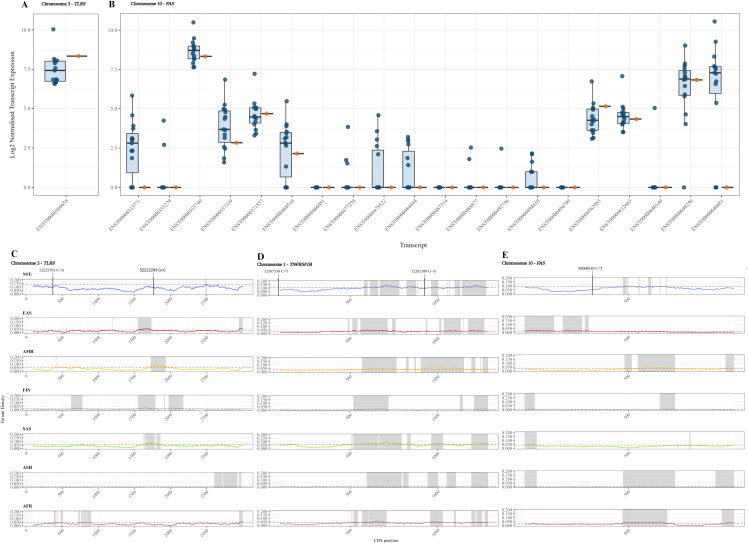
Differential transcript expression of *TLR9* **(A)** and *FAS* **(B)** in patients with MD compared with controls. In panels A and B, boxplots show the distribution of transcript expression in controls, the box represents the interquartile range, the central line indicates the median, and the whiskers represent the range of the data. Individual data points correspond to expression values for each control sample. Panels **(C–E)** show highly and lowly conserved sites for missense variants across different populations in *TLR9* **(C)** *TNFRSF1B* **(D)** and *FAS* **(E)** with selected mutations highlighted. NFE, Non-Finnish European, EAS, East Asian, AMR, Admixed American, FIN, Finnish European, SAS, South Asian, ASH, Ashkenazi Jew, AFR, Sub Saharan African.

To investigate functional consequences, we analysed bulk RNA-seq data from the individual carrying the two *TLR9* variants, along with sequenced controls. Differential expression analysis revealed a marked overexpression of *TLR9* in the individual carrying the variants (**Figure 2A**); however, it is not significant compared to controls.

Moreover, we retrieved RNA-seq data from one of the individuals with the *FAS* haplotype. Several transcripts showed lower expression in the MD sample compared with controls, with some transcripts not detected in the MD sample while present in controls. For transcripts that were detected, expression levels tended to be lower than the control average (**Figure 2B**).

### NF-κB pathway gene co-expression partners

To explore co-expression between different genes involved in the NF-κB pathway, we applied weighted gene co-expression network analysis. We found that the number of clusters in the controls is reduced from six to three in MD. Within the MD, we found that one cluster exhibits autoinflammatory co-expression, one similar to the Th2 immune response, and one undefined (**Supplementary Figures 19**–**22**). Two of the genes that carry rare variants were found to be co-expressed in the network. In the controls *FAS* was predicted to be co-expressed with *TLR10*, *WAC*, *MAP3K14*, and *TRAF5*; however, it was not found in the MD. On the other hand, *TNFRSF1B* was found in both the controls and MD. In the controls, *TNFRSF1B* is co-expressed with *HGF*, *MET*, *MYD88*, and *IL4*, whereas in MD, it is co-expressed with *IL1R1*.

### *TLR9*, *TNFRSF1B*, and *FAS* expression in the inner ear

None of the selected genes were significantly expressed in either the inner ear organoids or the SV datasets found in the gEAR portal (**Supplementary Tables 7**, **8**).

### Pathogenicity assessment of selected genes

The selected variants in *TLR9*, *TNFRSF1B*, and *FAS* fall within highly conserved regions of their respective CDS for the NFE population (**Figures 2C–E**).

No splicing elements were predicted with either *SpliceAI* or *Pangolin* for the selected variants in *TLR9*, *TNFRSF1B*, and *FAS*.

Human Splicing Finder Pro analysis of *TLR9* variant chr3:52222299 G>C found no significant impact on splicing signals. Conversely, the variant chr3:52223761 C>A showed a score increase from 46.68 to 73.82 (58.1%) for the donor site matrix, indicating the creation of a stronger donor splice site. The sequence change (GCAGGCAGG → GCAGTCAGG) resulted in the activation of a cryptic donor splice site, suggesting a potential alteration of normal splicing for transcript ENST00000360658 (**Table 5**).

**Table 5:** Splicing predictions according to Human Splice Finder Pro for chr3:52223761 C>A (*TLR9*) and chr1:12167150 C>T & chr1:12202100 G>A (*TNFRSF1B*).

Gene	*TLR9*	*TNFRSF1B*	
Variant	chr3:52223761 C>A	chr1:12167150 C>T	chr1:12202100 G>A
Strand	–	+	+
Algorithme/matrix	Donor site (matrix GT)	Donor site (matrix GT)	Acceptor site (matrix AG)
Position	chr3:52223765	chr1:12167146	chr1:12202090
Sequences	GCAGGCAGG>GCAGTCAGG	GCGGCGCAC>GCGGTGCAC	GCGCCCACTCGGAA>GCGCCCACTCAGAA
Variation	46.68>73.82 (58.14%)	42.51>69.65 (63.84%)	47.83>75.7 (58.27%)
Signal	New donor splice site	New donor splice site	New acceptor splice site
Interpretation	Activation of a cryptic donor site	Activation of a cryptic donor site	Activation of a cryptic acceptor site
Associated transcripts	ENST00000360658	ENST00000536782; ENST00000376259	ENST00000376259

For the *TNFRSF1B* variant chr1:12167150 C>T (affecting transcripts ENST00000492361.1, ENST00000536782.2, and ENST00000782365.1), HSF Pro identified an increase in the donor site matrix score from 42.51 to 69.65 (63.8%), indicating the creation of a stronger donor site at chr1:12167146 and activation of a cryptic donor splice site in transcripts ENST00000536782 and ENST00000376259. A significant alteration of auxiliary splicing regulatory elements was predicted, with an overall reduction in the ESE/ESS motif ratio by 4, including multiple broken ESE motifs and the creation of one new ESE ASF site (**Supplementary Table 9**). Furthermore, the variant chr1:12202100 G>A showed an increase in the acceptor site matrix score from 47.83 to 75.7 (58.3%) at chr1:12202090, indicating the creation of a stronger acceptor splice site in ENST00000376259.

For the *FAS*, neither the synonymous nor missense variant was predicted to have significant impact on core splice site signals detected in any of its transcripts. However, missense variant chr10:89008919 C>T was predicted to have a significant alteration of auxiliary splicing regulatory elements, with a reduction in the ESE/ESS motif ratio by five across multiple transcripts. The variant was associated with both the creation of new PESE ESE sites and the disruption of multiple ESE and ESS motifs (**Supplementary Table 10**).

### Protein models

**Figure 3:**
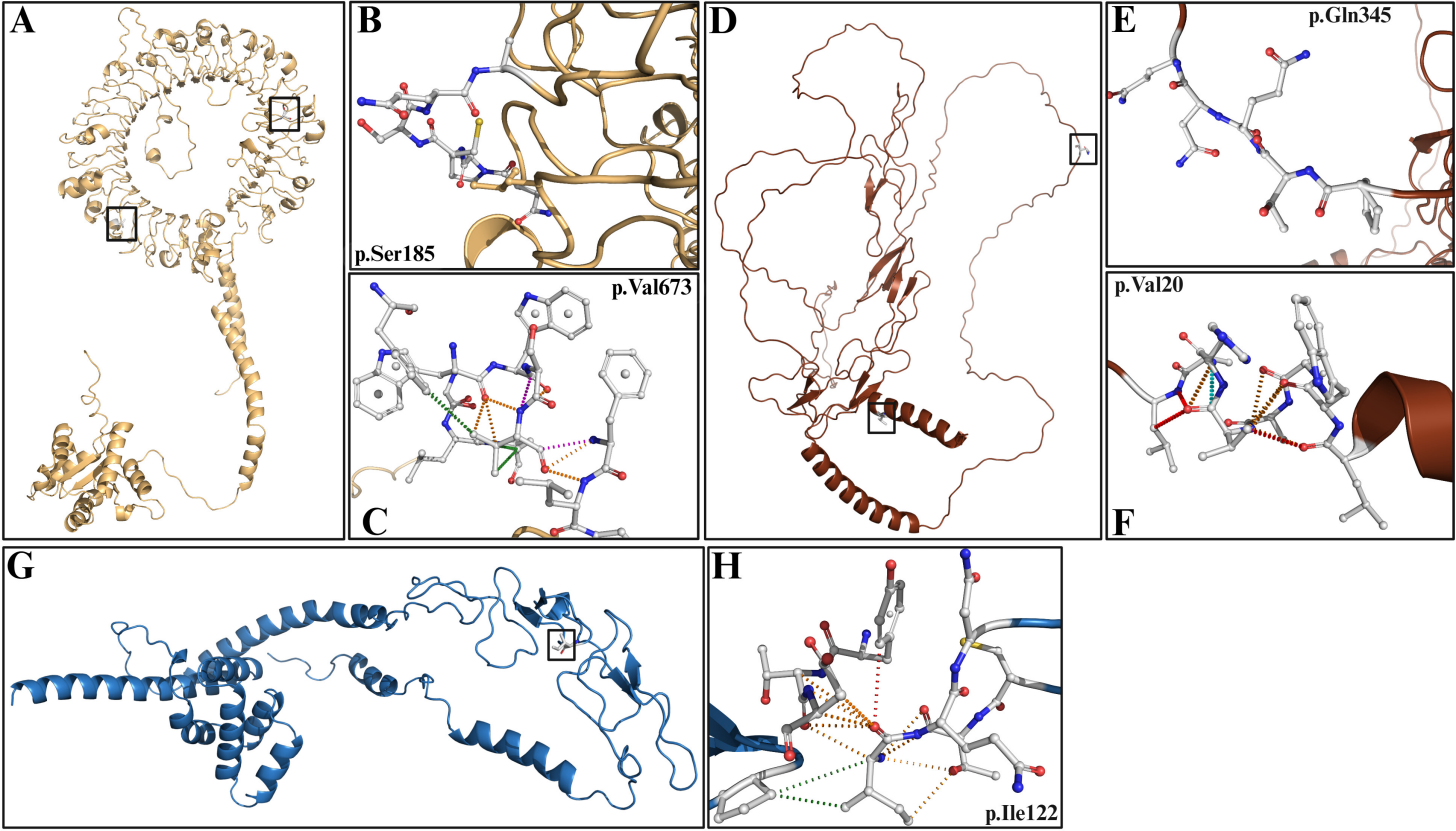
Protein models **(A–C)** Toll-like receptor 9 showing variants p.Ser185 and p.Val673. **(D–F)** Tumour necrosis factor receptor superfamily member 1B depicting variants p.Gln345 and p.Val20. **(G–H)** Tumour necrosis factor receptor superfamily member 6 showing variant p.Ile122.

The toll-like receptor 9 protein (*TLR9*) carrying both patient-derived variants (p.Arg185Ser and p.Leu673Val) was modelled to assess structural impact (**Figures 3A-C**). The p.Arg185Ser substitution produced a slight stabilising effect, with a predicted ΔΔG of 0.05 kcal mol^-^¹ (**Supplementary Table 11**). No additional atomic contacts or bonds were detected between the substituted residue and neighbouring amino acids. In contrast, the p.Leu673Val mutation was predicted to have a destabilising effect on the protein structure, with a ΔΔG of –1.15 kcal mol^-^¹. Dynamut2 predicted interactions with neighbouring amino acids in both the wild-type and mutant models. The mutant protein had significantly fewer bonds compared to its wild-type counterpart (**Supplementary Table 12**).

One MD patient carried two variants in the tumour necrosis factor receptor superfamily member 1B protein (*TNFRSF1B*), p.Ala20Val and p.Arg345Gln (**Figures 3D-F**). Both substitutions were predicted to destabilise the protein, with ΔΔG values of −0.45 kcal mol^-^¹ and −0.15 kcal mol^-^¹, respectively. For position 20, the mutant protein was predicted to form more interactions than the wild type (**Supplementary Table 13**). Conversely, for position 345, neither the wild-type nor the mutant protein model was predicted to form any bonds with neighbouring residues.

In the tumour necrosis factor receptor superfamily member 6 protein (*FAS*), a single amino acid substitution (p.Ile122Thr) was identified (**Figures 3G-H**). This substitution was predicted to be destabilising, with a ΔΔG of −0.7 kcal mol^-^¹. Dynamut2 predicted that the mutant protein model formed more interactions than the wild-type protein (**Supplementary Table 14**).

### Molecular docking with functional partners

The best docked models were selected considering the cluster centre as a representative middle point, the cluster size, and the lowest weighted energy score within each cluster (**Supplementary Tables 15**–**17**).

When the extracellular component of toll-like receptor 9 and the cytoplasmic region of tumour necrosis factor receptor superfamily member 1B were docked to their respective partners, they did not show any bonds being formed with our variants of interest (**Supplementary Figures 23**, **24****).**

**Figure 4:**
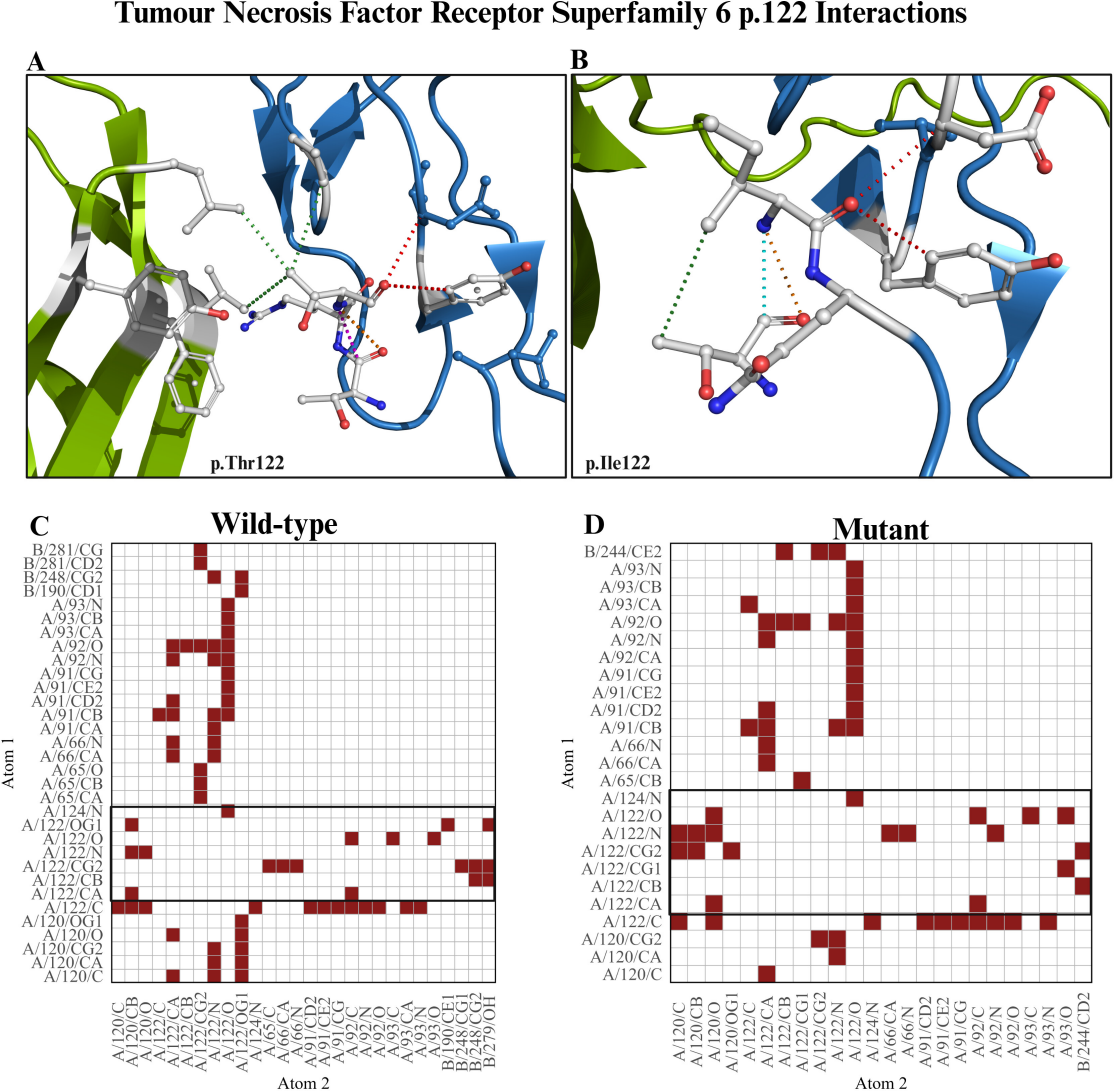
FAS-FASL docking models **(A)** Docking of wild-type extracellular component of tumour necrosis factor receptor superfamily member 6 (*FAS*) and wild-type extracellular component tumour necrosis factor ligand superfamily member 6 (*FASGL*) showing atomic interactions at p.Thr122. **(B)** Docking of mutant extracellular component *FAS* and wild-type extracellular component *FASGL* showing atomic interactions at p.Ile122. **(C)** Atom–atom interaction between wild-type *FAS*-*FASGL* docked model. **(D)** Atom–atom interaction between mutant *FAS*-*FASGL* docked model.

However, when the extracellular component of *FAS* protein was bound to the extracellular component of tumour necrosis factor ligand superfamily member 6 (*FASGL*), Arpeggio predicted that the wild-type model created a polar contact, two weak hydrogen bonds, three hydrophobic contacts, and one VDW clash, and 72 proximal mutually exclusive interactions (**Figure 4A**). In contrast, the p.Ile122 mutant docked protein loses two hydrophobic contacts and 14 proximal mutually exclusive interactions (**Figure 3B**). The predicted receptor-ligand interactions in the wild-type model are reduced from seven to two in the mutant docked model (**Figures 4C, D**).

Prodigy predicted that wild-type toll-like receptor 9 homodimers exhibit the strongest binding affinity, whereas the wild-type–mutant toll-like receptor 9 complex shows a reduced affinity, and the mutant homodimer largely restores binding strength (**Supplementary Table 18**). Although the overall interface size remains comparable across the three models, the mutation distributes interfacial interactions (ICs), decreasing polar and charged–polar contacts while increasing hydrophobic and mixed charged–apolar interactions, particularly in the mutant–mutant complex.

Both wild-type and mutant tumour necrosis factor receptor superfamily 6 docked complexes have identical binding affinities (**Supplementary Table 19**); however, it redistributes ICs, reducing polar interactions and increasing hydrophobic and mixed charged–hydrophobic contacts.

### *TLR9*, *TNFRSF1B*, and *FAS* homology across species

BLAST analysis of the queried sequences revealed strong matches to predicted mRNA sequences of *Pan troglodytes TLR9*, *TNFRSF1B*, and *FAS* genes (**Supplementary Tables 20**–**22**). The first query showed extensive similarity to *TLR9* sequences across multiple chimpanzee subspecies (*P*. *t. troglodytes*, *P*. *t*. *ellioti*, *P*. *t*. *verus*), with query coverage ranging from 32% to 54% and sequence identities above 99%, highlighting conserved regions among haplotypes. The E values were zero across all queries, indicating significant matches.

When multiple transcript variants of *TNFRSF1B* were aligned, it showed 6%–7% query coverage and high sequence identity (98.51%–98.55%) (*E*-value = 0).

Conversely, when *FAS* was matched, it showed 9%–10% query coverage and 98.21% sequence identity (*E*-value = 0), demonstrating conserved motifs across predicted mRNAs and miscellaneous RNA.

## Discussion

Patients with MD may have a systemic inflammation associated with different types of immune responses (41). Two responses have been identified in cross-sectional and longitudinal studies: a Th2-mediated response associated with an increase in granulocytes (13, 17, 18) and a monocyte-driven autoinflammatory response (13, 15, 18). This inflammatory response can be modulated by genetic and environmental factors and determine the clinical course and outcome in MD patients. Our study highlights genes with an overrepresentation of ultra-rare variants in the NF-κB pathway genes when we compared the number of missense and synonymous variants in the CDS for the same genes in individuals with MD. Rare variants are known to have large effect sizes at the individual level, which may contribute to extreme phenotypes. This suggests that rare variants in innate immune genes could play a key role in shaping the heterogeneity of the phenotype observed in MD. Although a burden of synonymous variants was also observed in some genes such as *TLR9*, none of the synonymous variants were present in the same individuals carrying missense variants forming a haplotype. These findings suggest that ultra-rare missense variants with a large effect size could mediate the proinflammatory response observed in patients with an autoinflammatory immunophenotype.

Common variants in *TLR9* have been previously associated with autoimmune/autoinflammatory disorders across different populations. In a 2013 study (70), using a non-Finnish European cohort, it was found that *TLR9* does increase the risk of systemic lupus erythematosus (SLE). Moreover, patients with SLE and both homozygous and heterozygous T alleles in rs352140 had an increased likelihood of more severe kidney and immunologic disorders and increased levels of anti-dsDNA antibodies.

Two common variants (rs352139 and rs352140) in *TLR9* were found to be statistically more common in a Han Chinese cohort with SLE and nephritis than in healthy controls (71, 72). Moreover, the homozygous TT genotype of rs352139 has been associated with type 1 diabetes in a Han Chinese cohort (73).

Computational docking and structural analyses of the toll-like receptor 9 mutant dimer are found in the extracellular component of the receptor and predict that the two variants do not interact with each other in either the wild-type–wild-type, wild-type–mutant and mutant–mutant configuration. However, the mutations may increase hydrophobic interactions, potentially stabilising receptor dimerisation and enhancing downstream signalling.

Considering previous studies, we cannot rule out that specific common variants in *TLR9* may be more strongly associated with autoimmune and autoinflammatory disorders unrelated to MD, such as rheumatoid arthritis or Raynaud syndrome, as observed in *Patient 1*. Mutations in *TLR9* have previously been associated with rheumatoid arthritis. A 2011 study reported that the rs187084 variant in *TLR9* likely confers susceptibility to rheumatoid arthritis in the Turkish population (74). This supports the idea that MD has two “native” immune phenotypes and one that is associated with several autoimmune disorders.

A 2017 study found that the *TNF* rs1800630 and *TNFRSF1B* rs1061624 variants are associated with an increased risk of SNHL in elderly Japanese individuals, suggesting that TNF-α–mediated pathways possibly play a role in the development of age-related hearing loss (75). In the present study, we found that both protein models are destabilising in their monomeric forms; however, *TNFRSF1B* variants do not interact with their ligands. Moreover, both chr1:12167150 C>T and chr1:12202100 G>A variants show a high likelihood of creating splice sites, potentially rendering non-functional protein products.

Similarly, two common variants in the *FAS* (rs1468063 and rs2862833) identified in Chinese workers were associated with a reduced risk of noise-induced hearing loss (76). In the same study, noise-exposed rats exhibited increased *FAS* expression along with elevated hearing thresholds, oxidative stress, and DNA damage and with a decrease in microRNA *let-7e*. The authors suggested that *FAS* activation is mediated by reactive oxygen species and contributes to noise-induced hearing loss.

The *FAS* variant chr10:89003139 G>A was found replicated in two individuals from the AMR cohort (32). Functional studies will be required to determine how this variant affects the RNA transcript in the affected individuals.

In this study, we found that the *FAS*–*FASGL* docked model predicts interaction in the wild type but loses it in the mutant amino acid. This is particularly interesting, as this position has already been experimentally identified as a key contact point between the two dimers (77, 78). The loss of these contacts could be detrimental to dimer function, potentially leading to an abnormal immune response. Furthermore, the *FAS*–*FASGL* dimer was predicted to be shifting the complex toward a more hydrophobic and charged–apolar-driven interface rather than polar interactions, which together offset the loss and stabilize the complex.

Genes in the NF-κB signalling pathway have been implicated in MD. Common variants in *NFKB1* have been associated with faster progression of SNHL in unilateral MD, while other variants are linked to bilateral MD through eQTL-mediated upregulation of the TWEAK pathway in PBMCs (25, 26). Moreover, TWEAK is secreted by intermediate cells, whereas its receptor is expressed in marginal and spindle cells of the SV (27).

Taken together, these findings, together with the absence of reported autoimmune/autoinflammatory comorbidities in patients carrying variants in NF-κB pathway genes such as *TNFRSF1B* and *FAS*, suggest that these individuals may fall within one previously described immunophenotype, although validation would require supporting cytokine functional data.

Overall, these results indicate that the query sequences correspond to highly conserved regions of key immune-related genes in *Pan troglodytes*, with multiple transcript variants represented. However, our results indicate that the variants are in low-density regions in the NFE population.

This study has several limitations. Datasets include MD cases from multiple populations, introducing heterogeneity. Analyses rely on external reference populations, which may cause ancestry-related or technical confounding, and statistical power is limited for rare variant genotype-phenotype links. Computational predictions of splicing and protein structural effects are hypothesis-generating and require experimental validation with RNA assays or protein functional studies. The absence of direct experimental validation limits the ability to confirm the functional impact of the identified variants. Notably, while SpliceAI and Pangolin did not predict splice site changes for any of the variants, HSF Pro identified cryptic splice site activation and alterations in auxiliary splicing elements for specific variants, highlighting that motif-based analysis can reveal subtle splicing effects not captured by deep learning models. The absence of direct experimental validation limits the ability to confirm the functional impact of the identified variants.

## Conclusions

In conclusion, there is a burden of ultrarare missense variants in NF-κB pathway genes, including *FAS*, *TLR9*, and *TNFRSF1B.* These data support that MD may comprise different immune phenotypes, with some characterised by autoimmune condition comorbidities. Importantly, this study highlights the potential of genetic profiling for MD patient stratification and to improve the understanding of the disease mechanisms, particularly specific immune pathways. Future studies are needed to validate these associations in larger cohorts and to explore how these variants influence disease onset, severity, and response to treatment.

## Data Availability

The TLR9 (chr3:52222299 G>C; chr3:52223761 C>A), TNFRSF1B (chr1:12167150 C>T; chr1:12202100 G>A), and FAS (chr10:89003139 G>A; chr10:89008919 C>T) variants identified in this study have been deposited in ClinVar under submission number SUB15898535.
