# Supplementary material for: Burden of novel and ultra-rare missense variants in the NF-κB pathway genes associated to Ménière’s disease
Source: Front Immunol. 2026 May 15;17:1789753. doi: 10.3389/fimmu.2026.1789753 (PMC13218865; doi:10.3389/fimmu.2026.1789753)

**Table of Contents**

[**Supplementary Figure 1.-** Novel variant chr1:206479081 C<A in the *IKBKE* gene found in a MD patient. 3](#_Toc224128876)

[**Supplementary Figure 2.-** Novel variant chr4:153704469 C<G in the *TLR2* gene found in a MD patient. 4](#_Toc224128877)

[**Supplementary Figure 3.-** Novel variant chr4:153704469 C<G in the *TLR2* gene found in a MD patient. 5](#_Toc224128878)

[**Supplementary Figure 4.-** Novel variant chr9:117712631 C<A in the *TLR4* gene found in a MD patient. 6](#_Toc224128879)

[**Supplementary Figure 5.-** Novel variant chr9:117714265 A<G in the *TLR4* gene found in a MD patient. 7](#_Toc224128880)

[**Supplementary Figure 6.-** Novel variant chr1:223111641 C<T in the *TLR5* gene found in a MD patient. 8](#_Toc224128881)

[**Supplementary Figure 7.-** Novel variant chr3:52221977 G<A in the *TLR9* gene found in a MD patient. 9](#_Toc224128882)

[**Supplementary Figure 8.-** Novel variant chr1:12167150 C<T in the *TNFRSF1B* gene found in a MD patient. 10](#_Toc224128883)

[**Supplementary Figure 9.-** Novel variant chr1:12202091 C<A in the *TNFRSF1B* gene found in a MD patient. 11](#_Toc224128884)

[**Supplementary Figure 10.-** Novel variant chr17:7549486 G<C in the *TNFSF12* gene found in a MD patient. 12](#_Toc224128885)

[**Supplementary Figure 11.-** Novel variant chr9:136900486 G<A in the *TRAF2* gene found in a MD patient. 13](#_Toc224128886)

[**Supplementary Figure 13.-** Novel variant chr9:136925701 G<A in the *TRAF2* gene found in a MD patient. 15](#_Toc224128887)

[**Supplementary Figure 14.-** Novel variant chr9:136925897 T<C in the *TRAF2* gene found in a MD patient. 16](#_Toc224128888)

[**Supplementary Figure 15.-** Patient 1 air conducted hearing threshold audiogram with bilatera synchornic SNHL. Left ear represented in blue; right in red. 17](#_Toc224128889)

[**Supplementary Figure 16.-** Patient 2 air conducted hearing threshold audiogram with bilateral synchronic SNHL’. Left ear represented in blue; right in red. 18](#_Toc224128890)

[**Supplementary Figure 17.-** Patient 3 air conducted hearing threshold audiogram with unilateral SNHL in right ear. Left ear represented in blue; right in red. 19](#_Toc224128891)

[**Supplementary Figure 18.-** Patient 4 air conducted hearing threshold audiogram with permanenert bilateral hearing loss. Left ear represented in blue; right in red. 20](#_Toc224128892)

[**Supplementary Figure 19.-** Predicted clusters in single-cell RNA sequencing controls 21](#_Toc224128893)

[**Supplementary Figure 20.-** Co-expression of NF-κB pathaway genes in the controls single-cell RNA sequecing predicted cluster 22](#_Toc224128894)

[**Supplementary Figure 21.-** Predicted clusters in single-cell RNA sequencing Menire Disease 23](#_Toc224128895)

[**Supplementary Figure 22.-** Co-expression of NF-κB pathaway genes in the Menire Disease single-cell RNA sequecing predicted cluster 24](#_Toc224128896)

[**Supplementary Figure 23.-** Docked *TLR9*-*TLR9* models. (A) Wild-type – wild-type *TLR9* dimer. (B) Wild-type – mutant *TLR9* dimer. (C) Mutant – mutant *TLR9* dimer. 25](#_Toc224128897)

[**Supplementary Figure 24.-** Docked *TNFRSFB*-*TRAF2* models. (A) Wild-type – wild-type *TNFRSFB*-*TRAF2*. (B) Wild-type – mutant *TNFRSFB*-*TRAF2*. 26](#_Toc224128898)

###
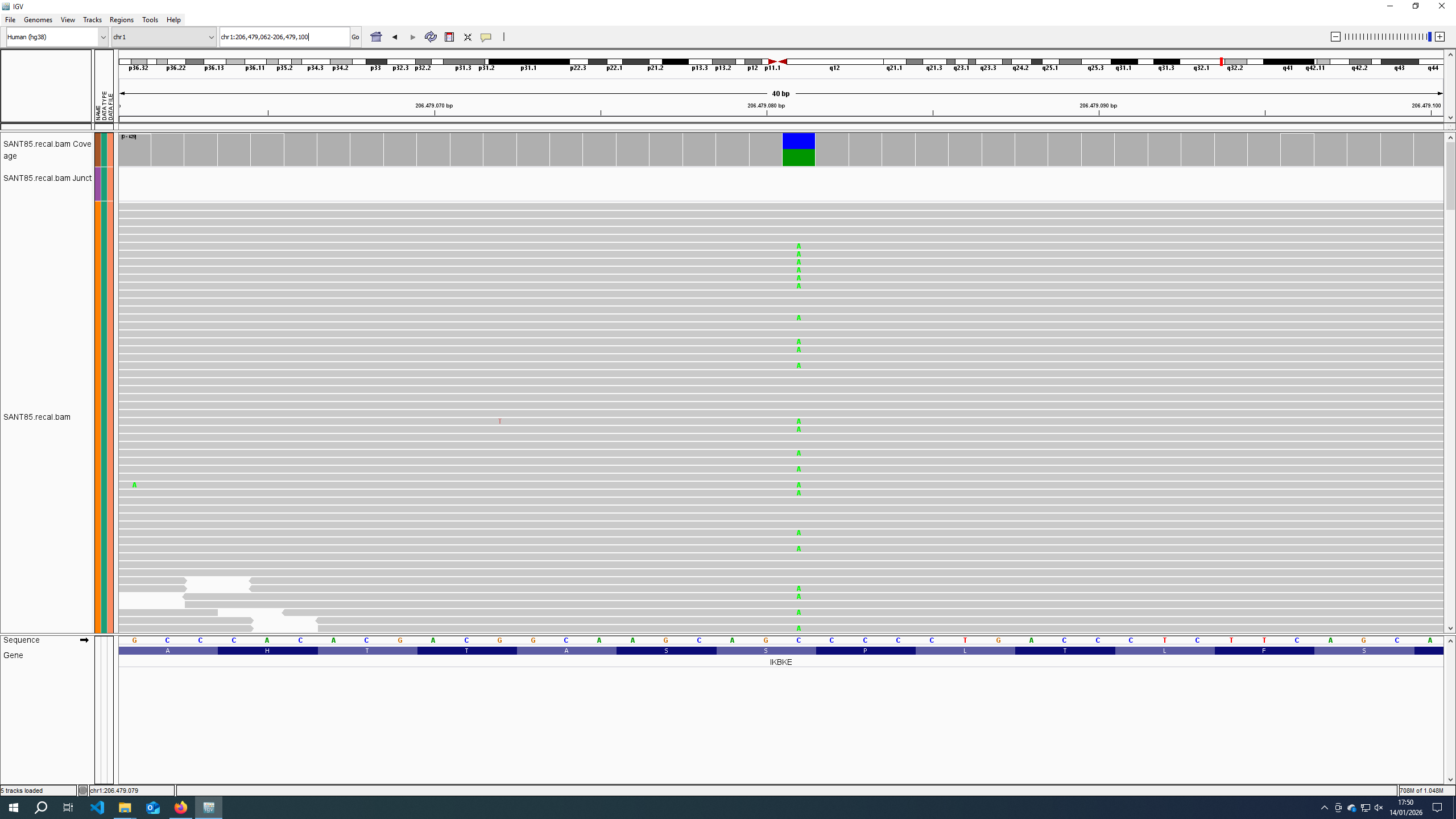
**Supplementary Figure 1.-** Novel variant chr1:206479081 C<A in the *IKBKE* gene found in a MD patient.

### **Supplementary Figure 2.-** Novel variant chr4:153704469 C<G in the *TLR2* gene found in a MD patient.

**
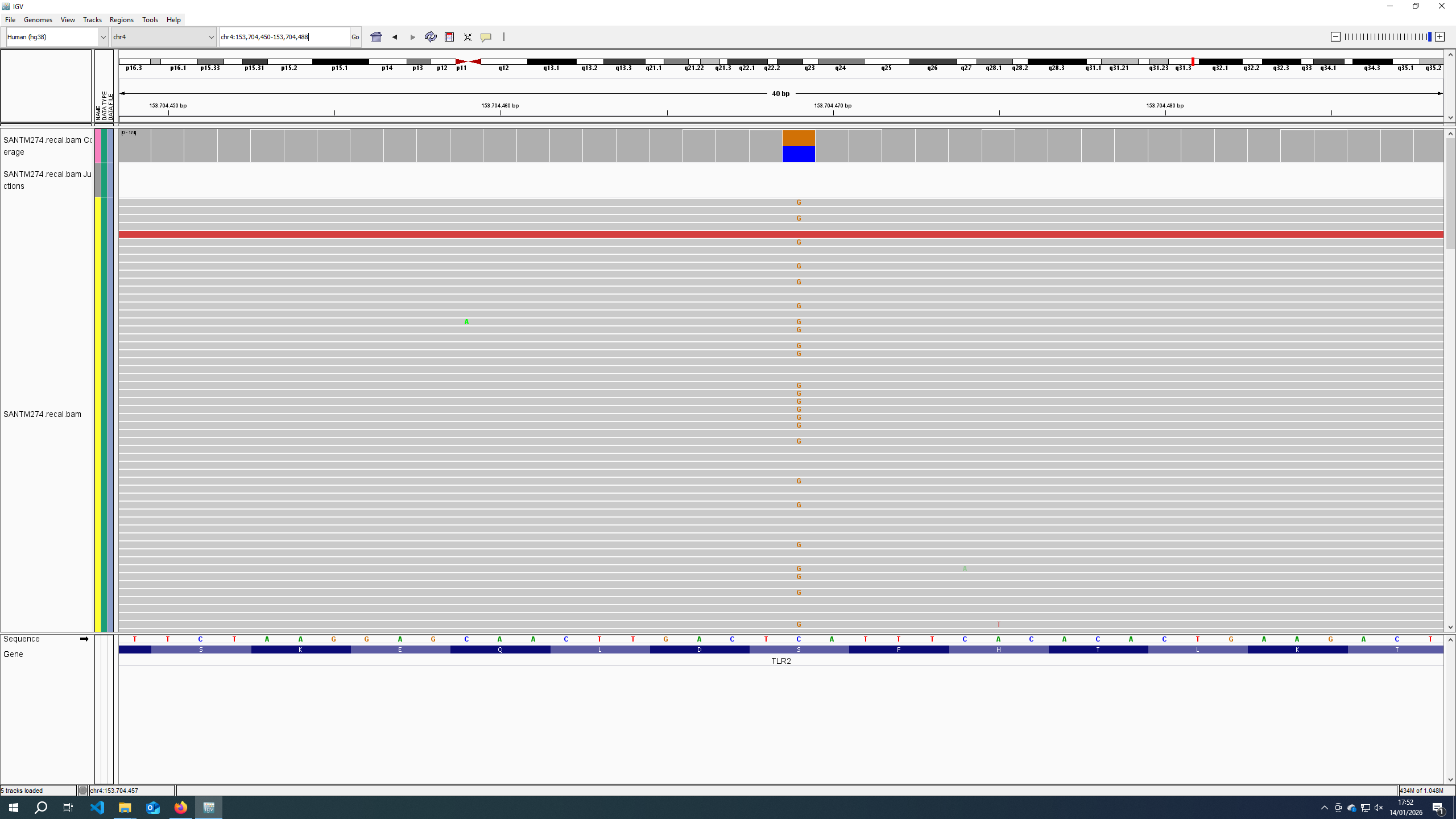
**

### **Supplementary Figure 3.-** Novel variant chr4:153704469 C<G in the *TLR2* gene found in a MD patient.

**
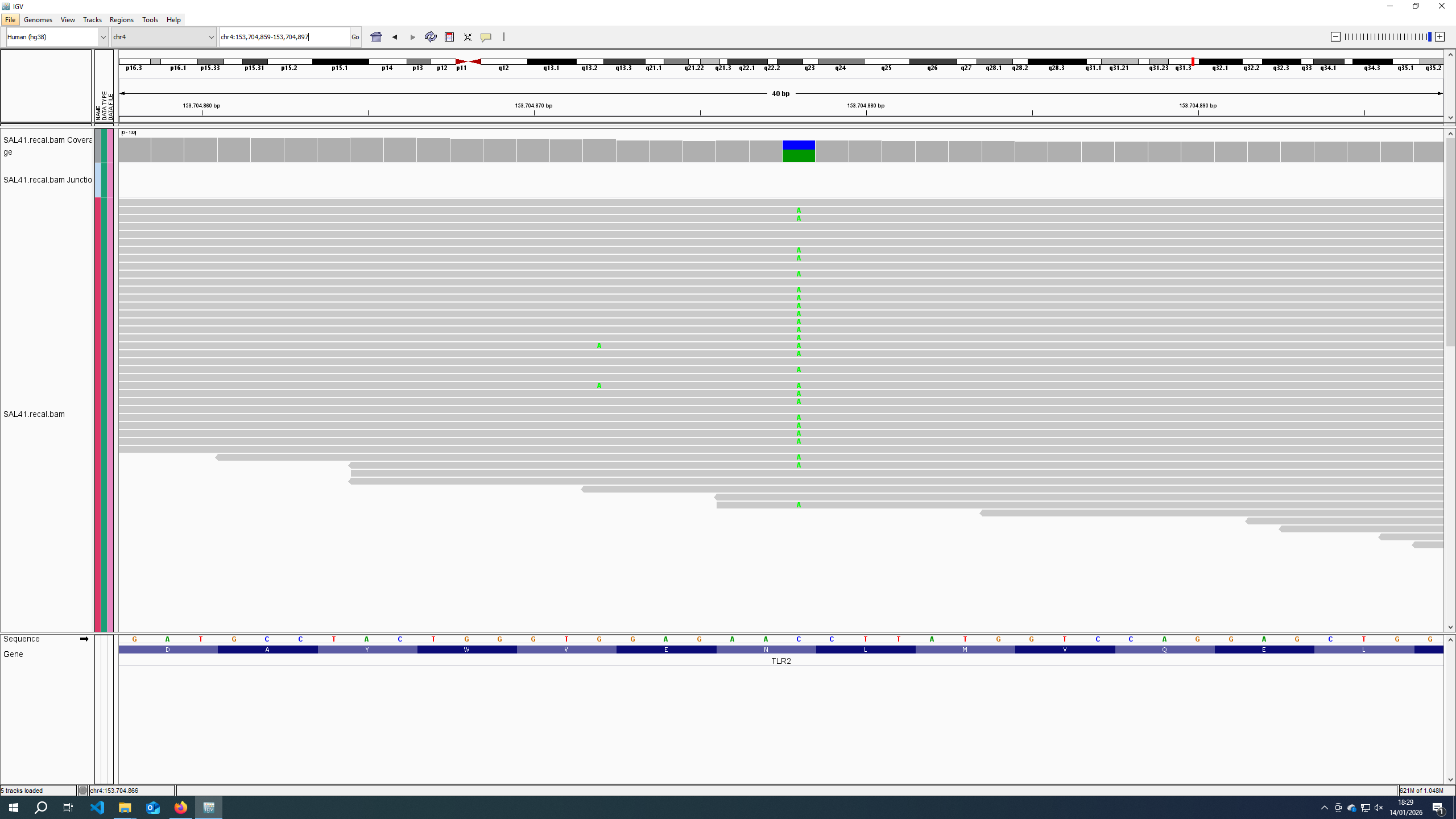
**

### **Supplementary Figure 4.-** Novel variant chr9:117712631 C<A in the *TLR4* gene found in a MD patient.

**
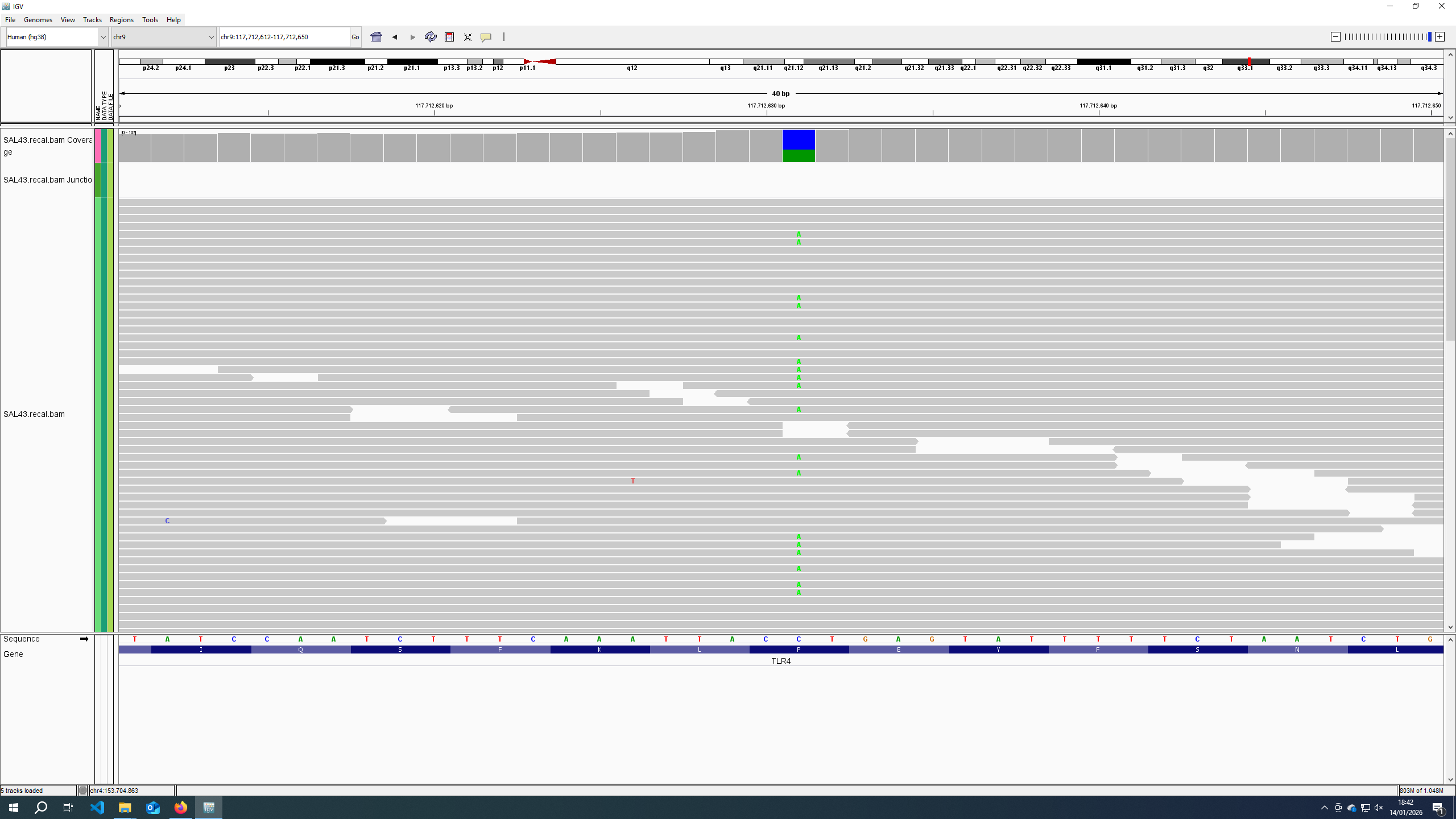
**

### **Supplementary Figure 5.-** Novel variant chr9:117714265 A<G in the *TLR4* gene found in a MD patient.


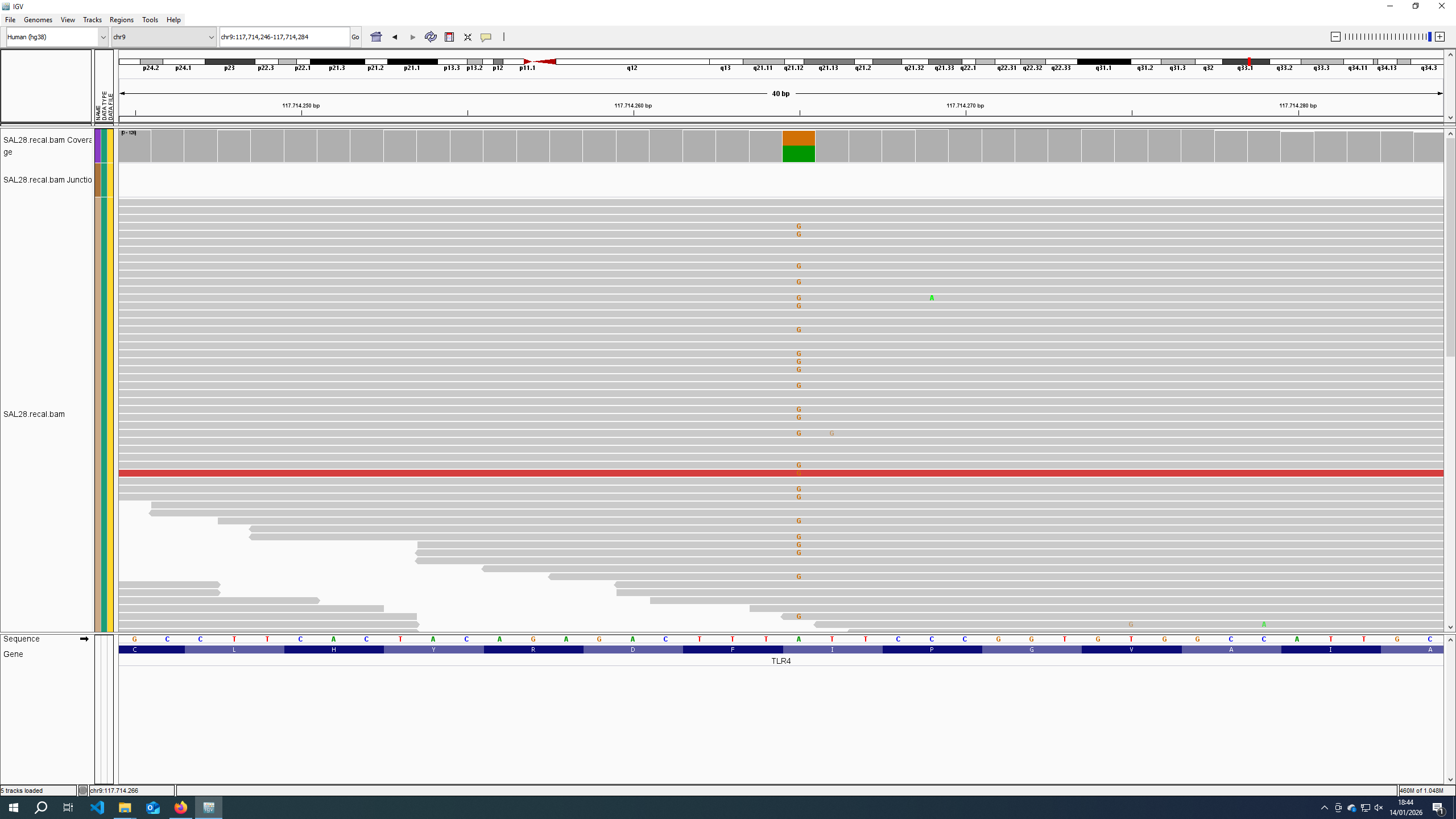


### **Supplementary Figure 6.-** Novel variant chr1:223111641 C<T in the *TLR5* gene found in a MD patient.


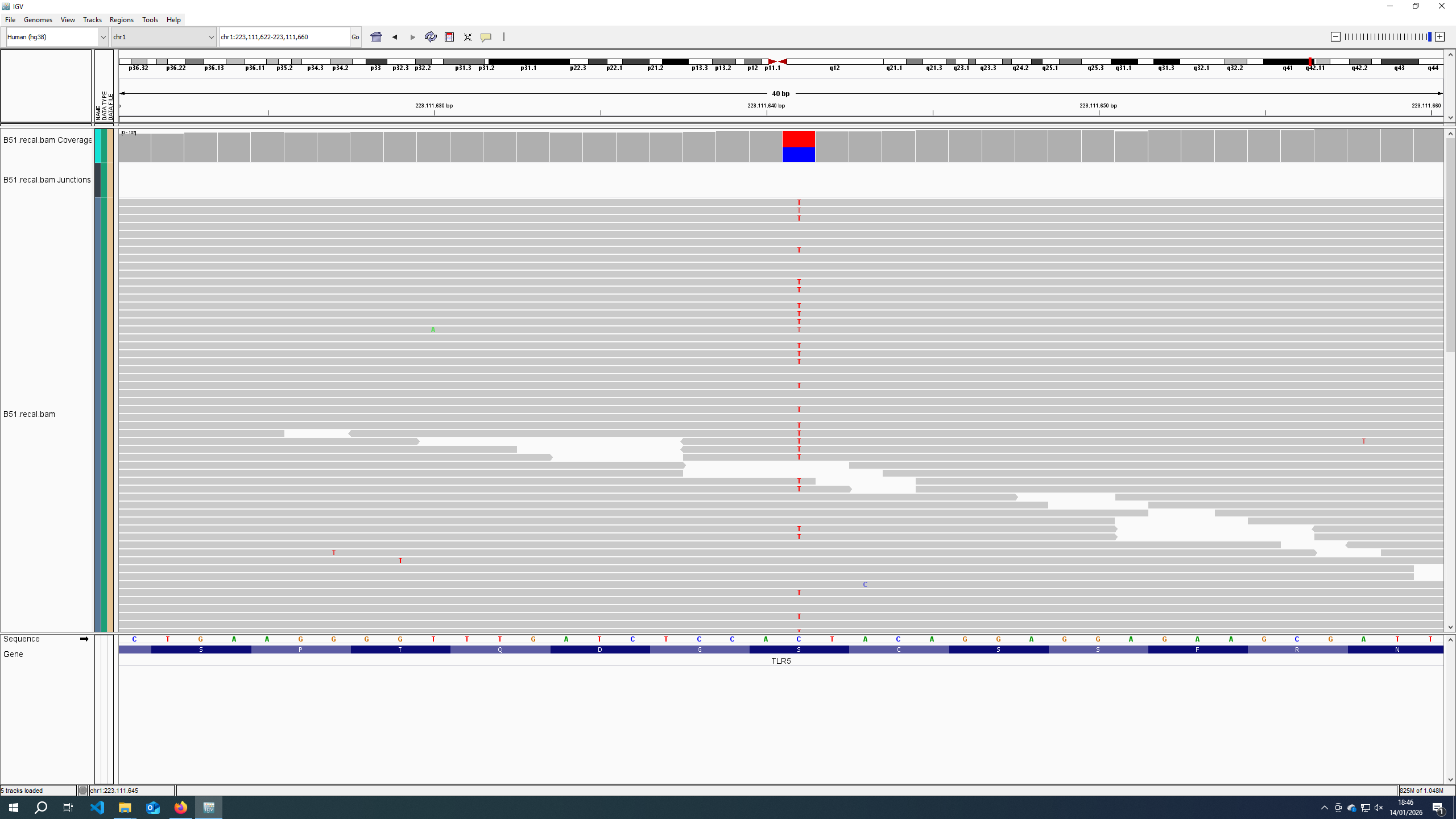


### **Supplementary Figure 7.-** Novel variant chr3:52221977 G<A in the *TLR9* gene found in a MD patient.


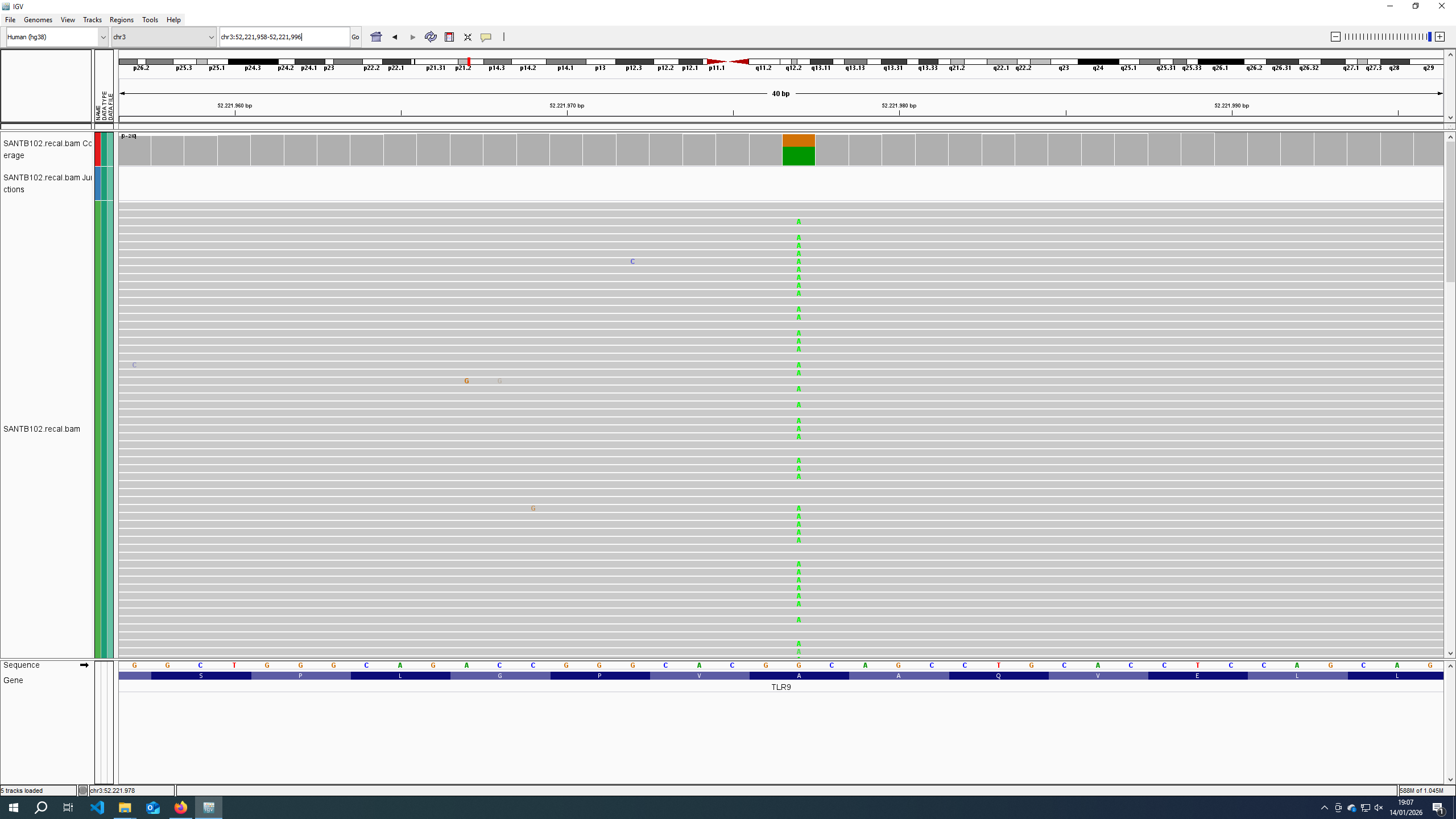


###
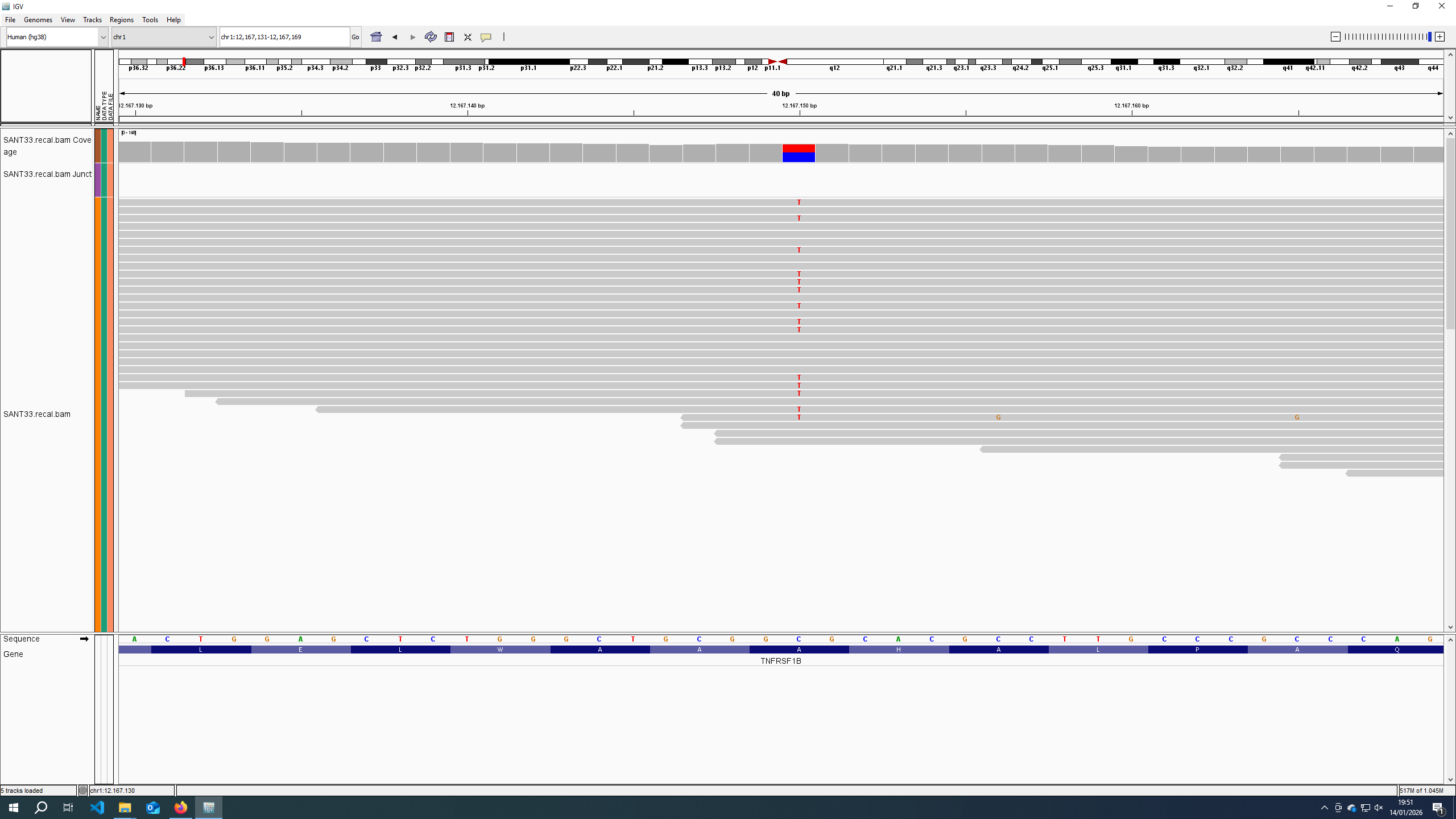
**Supplementary Figure 8.-** Novel variant chr1:12167150 C<T in the *TNFRSF1B* gene found in a MD patient.

### **Supplementary Figure 9.-** Novel variant chr1:12202091 C<A in the *TNFRSF1B* gene found in a MD patient.


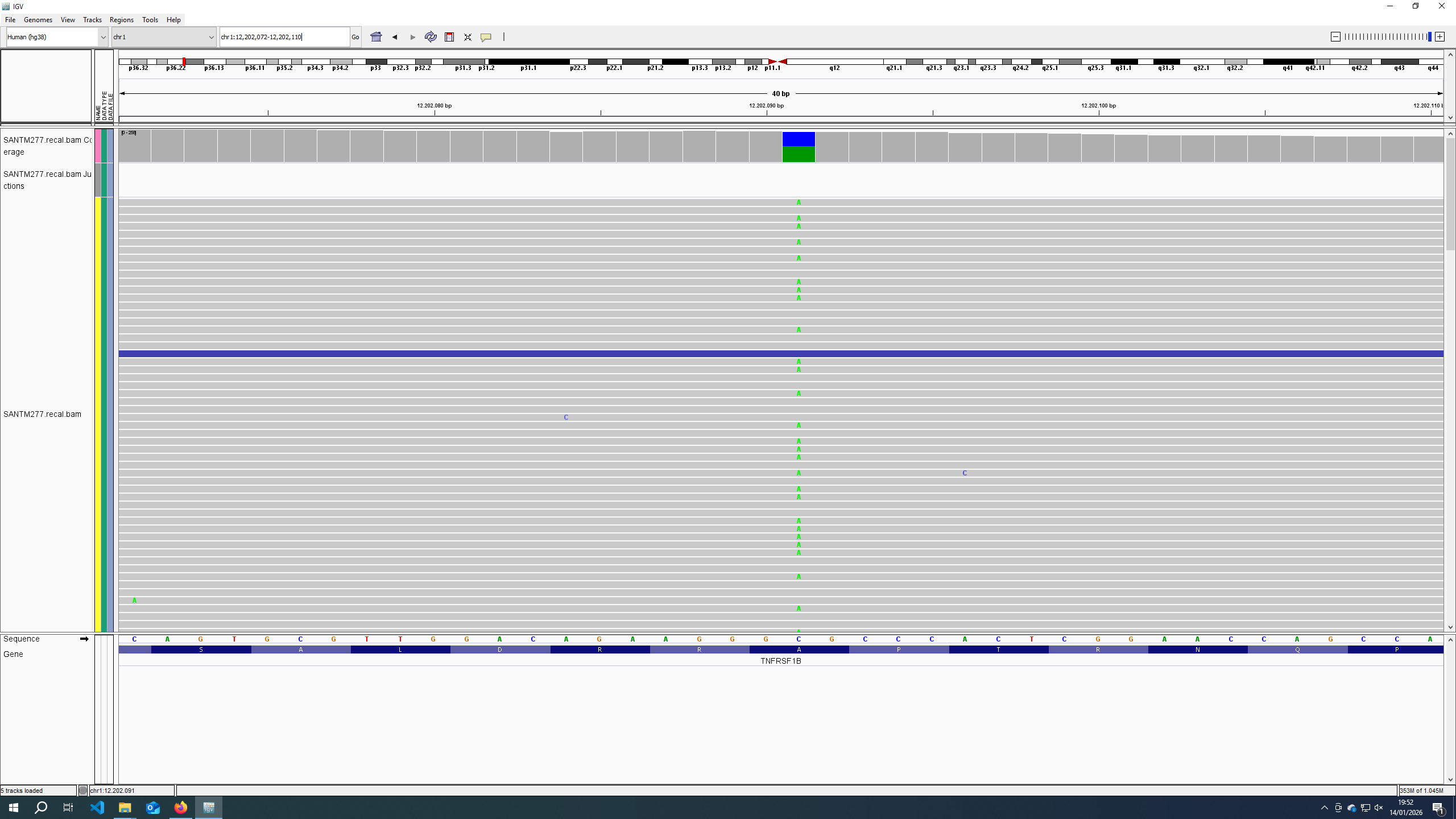


### **Supplementary Figure 10.-** Novel variant chr17:7549486 G<C in the *TNFSF12* gene found in a MD patient.


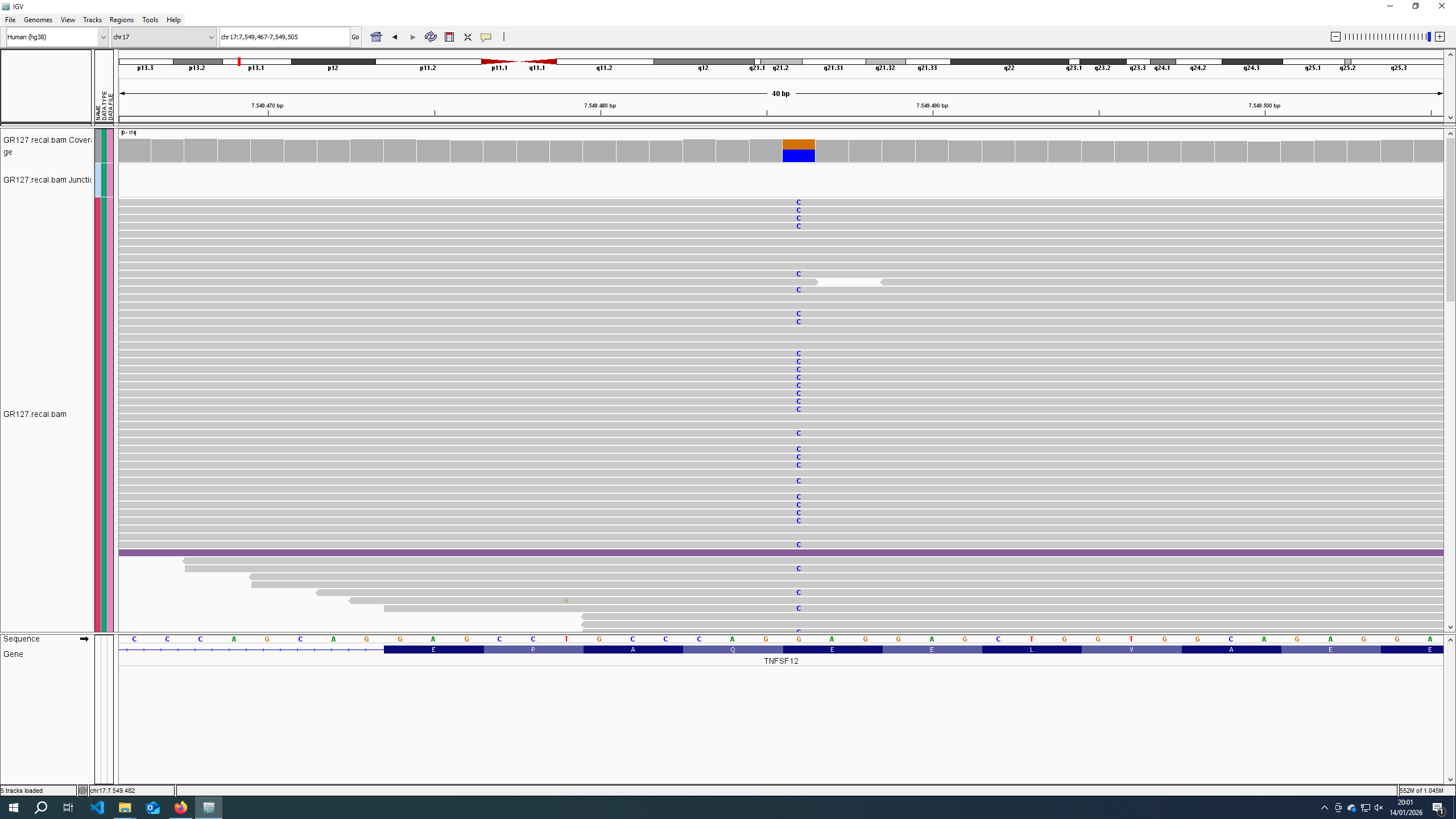


### **Supplementary Figure 11.-** Novel variant chr9:136900486 G<A in the *TRAF2* gene found in a MD patient.


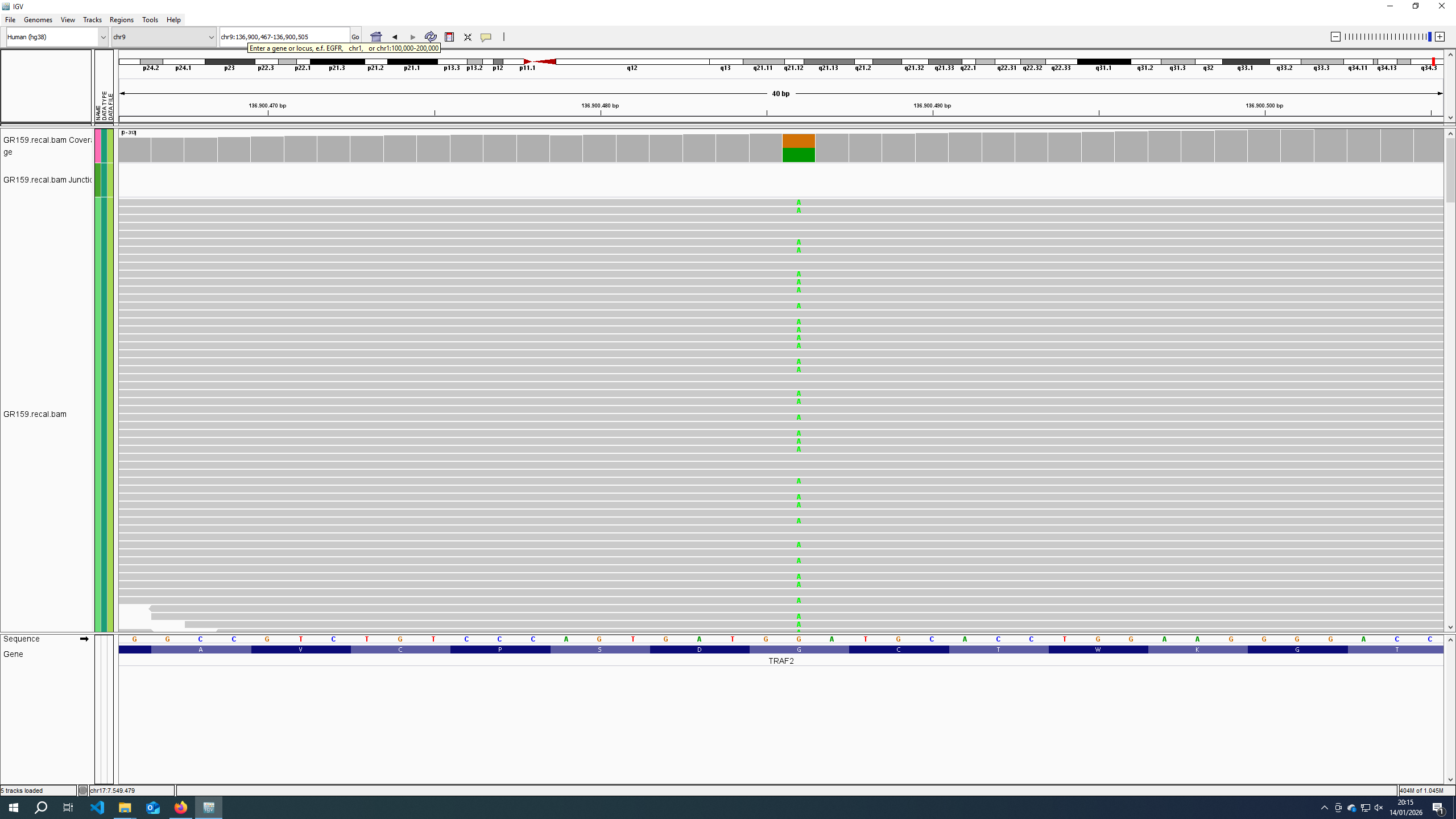


**Supplementary Figure 12.-** Novel variant chr9:136920408 A<G in the *TRAF2* gene found in a MD patient.


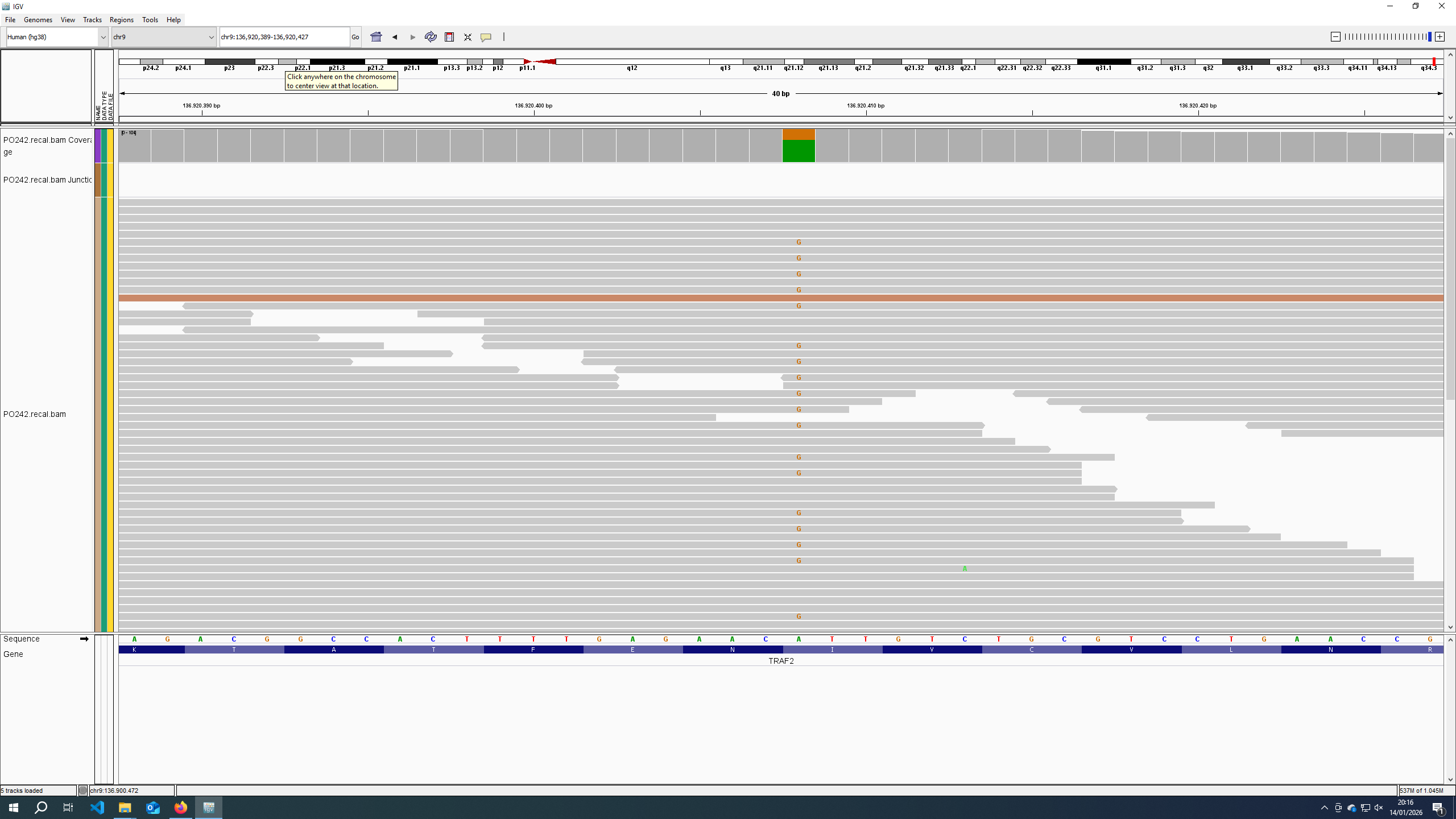


### **Supplementary Figure 13.-** Novel variant chr9:136925701 G<A in the *TRAF2* gene found in a MD patient.


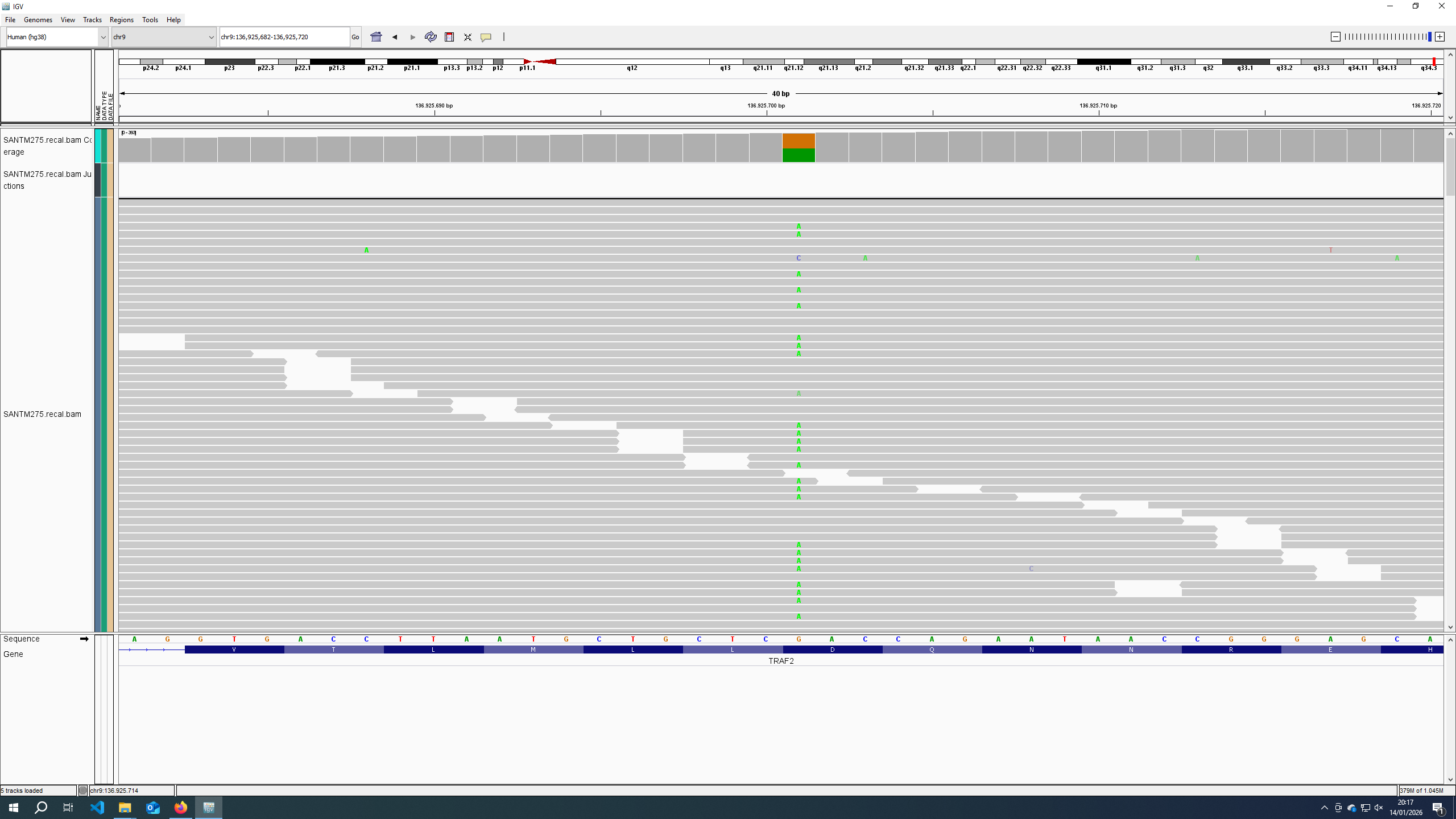


### **
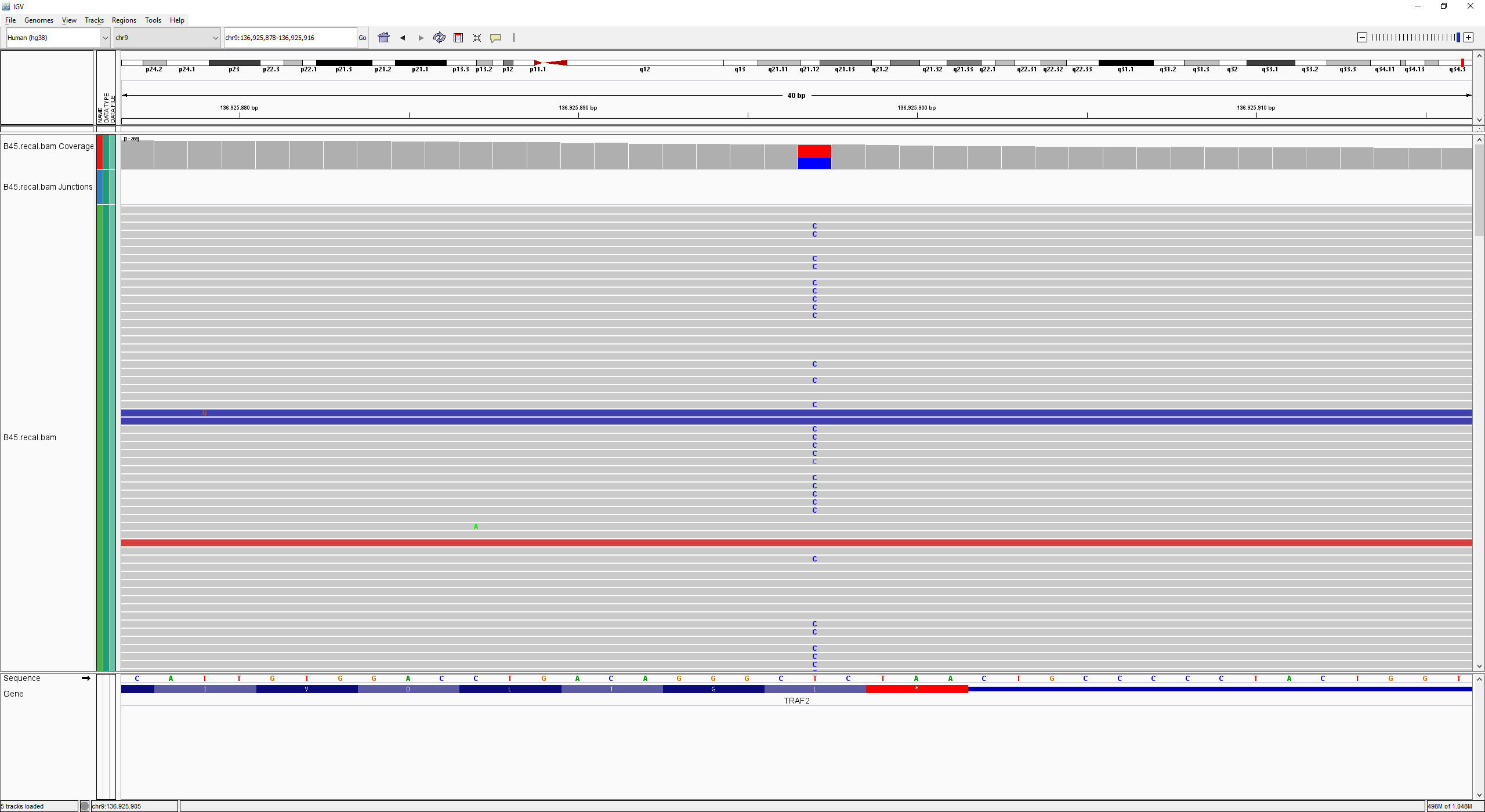
Supplementary Figure 14.-** Novel variant chr9:136925897 T<C in the *TRAF2* gene found in a MD patient.

#
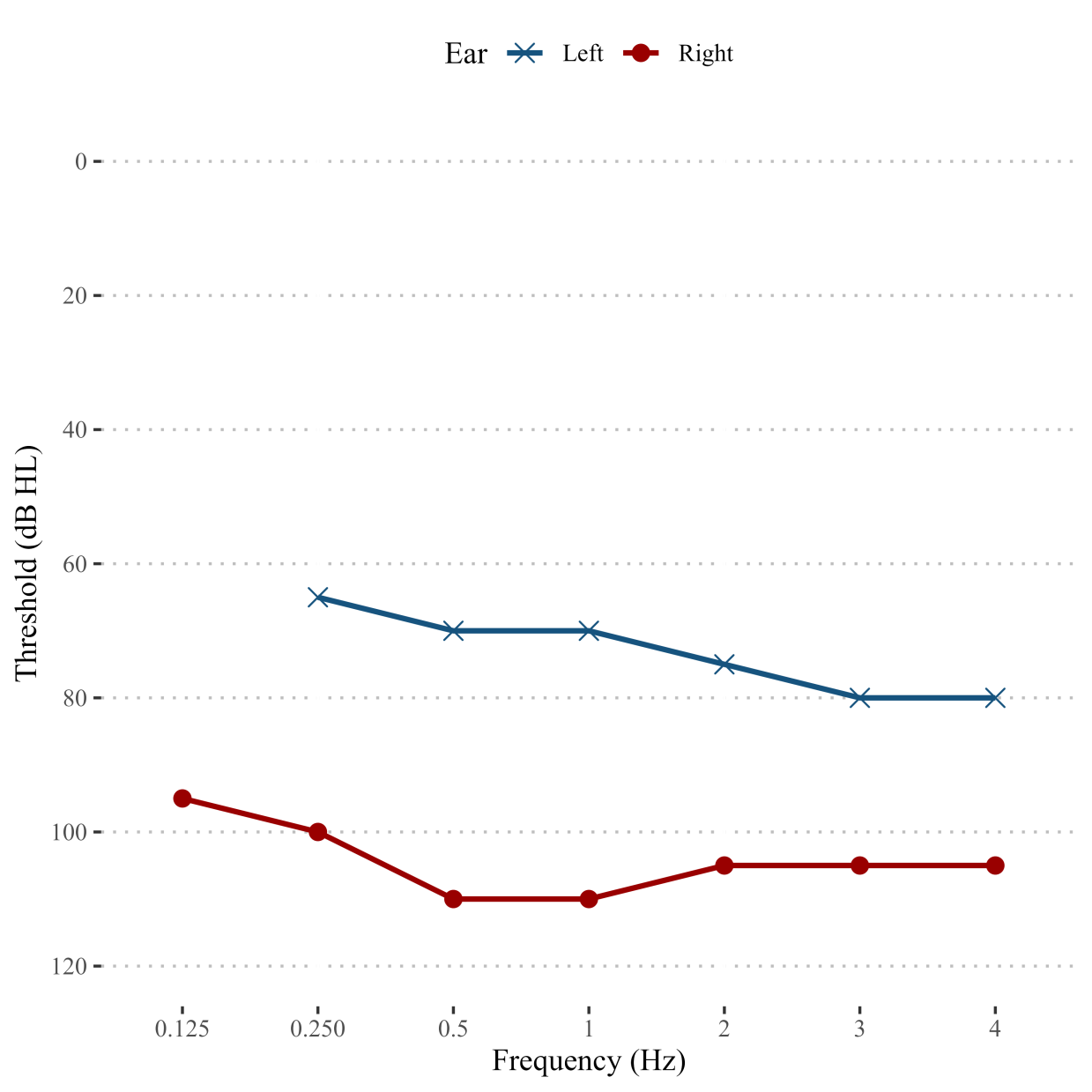
**Supplementary Figure 15.-** Patient 1 air conducted hearing threshold audiogram with bilateral synchronic SNHL. Left ear represented in blue; right in red. Right Ear PTA ≈108 dB HL, Left Ear PTA ≈72 dB HL. PTA = Pure Tone Average, dB HL = decibel hearing level.

#
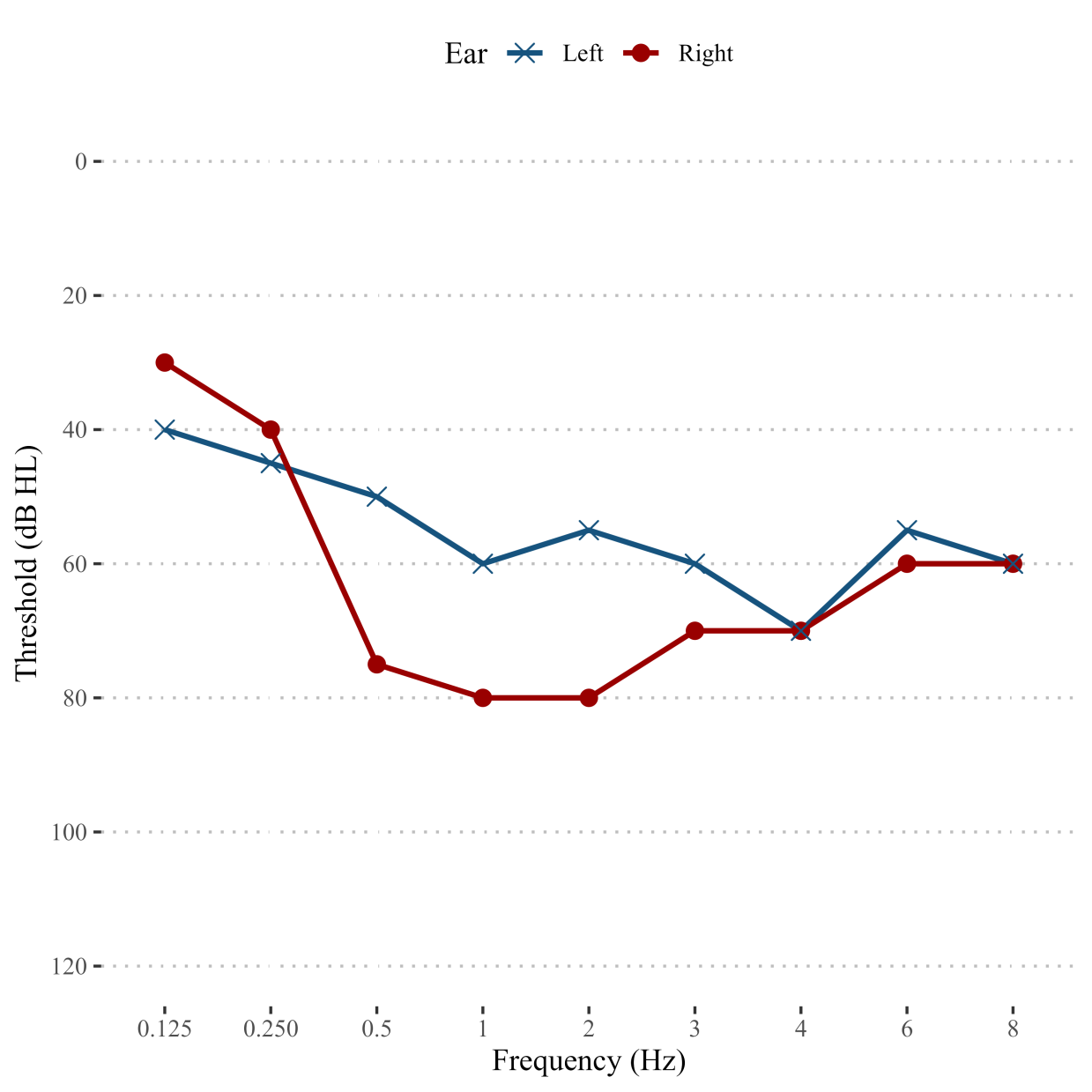
**Supplementary Figure 16.-** Patient 2 air conducted hearing threshold audiogram with bilateral synchronic SNHL. Left ear represented in blue; right in red. Right Ear PTA ≈78 dB HL, Left Ear PTA ≈55 dB HL. PTA = Pure Tone Average, dB HL = decibel hearing level.

#
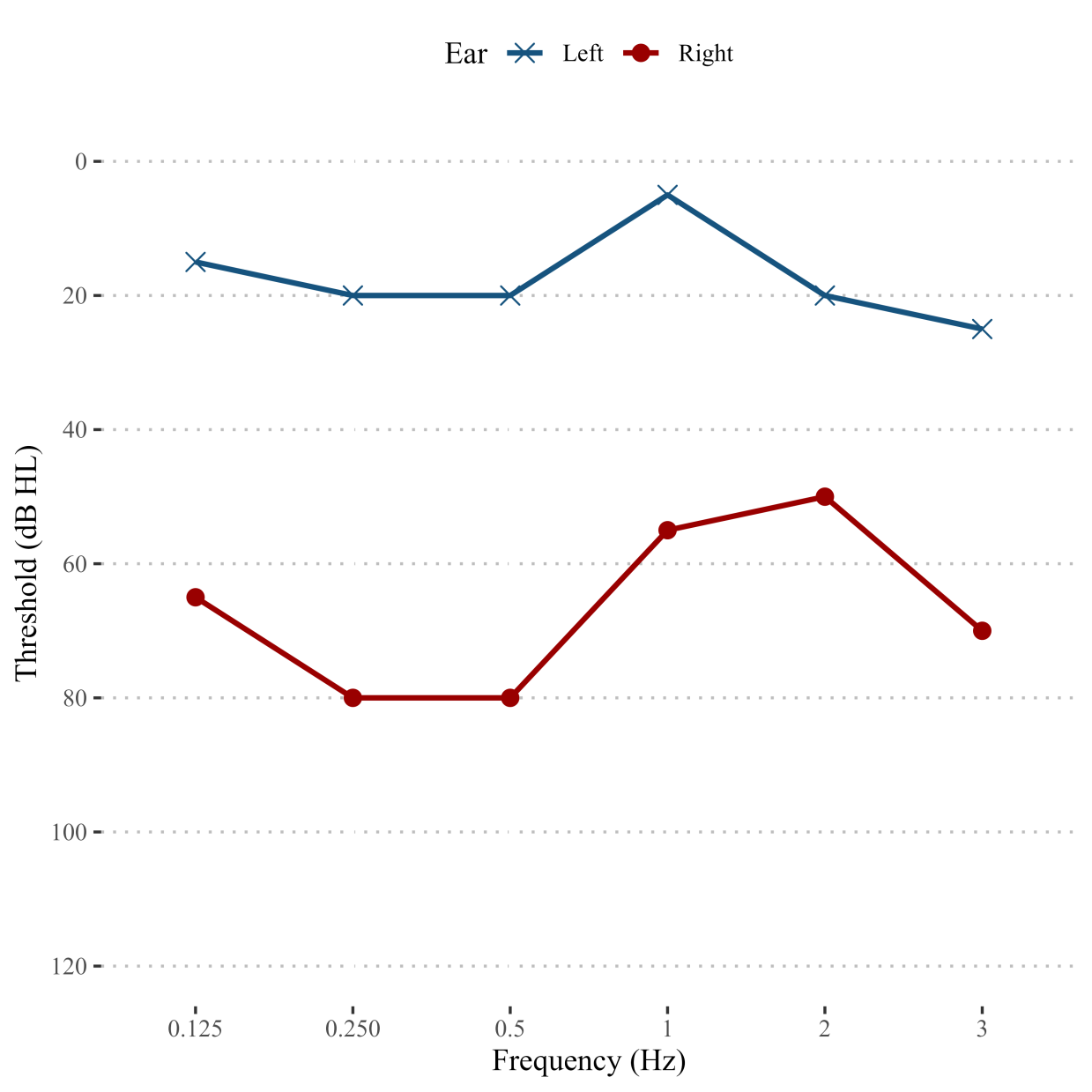
**Supplementary Figure 17.-** Patient 3 air conducted hearing threshold audiogram with unilateral SNHL in right ear. Left ear represented in blue; right in red. Right Ear PTA ≈62 dB HL, Left Ear PTA ≈15 dB HL. PTA = Pure Tone Average, dB HL = decibel hearing level.

# **
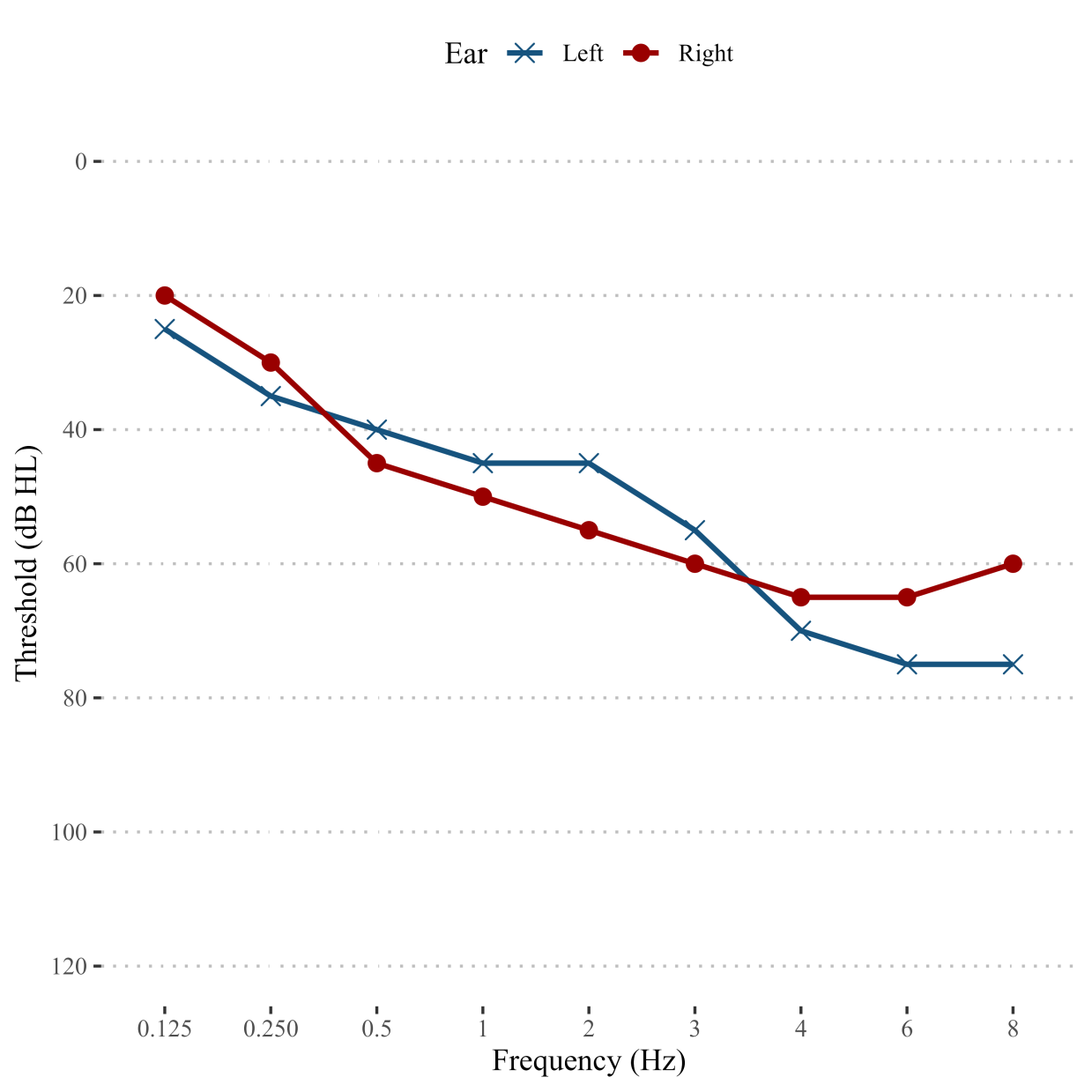
Supplementary Figure 18.-** Patient 4 air conducted hearing threshold audiogram with permanenert bilateral hearing loss. Left ear represented in blue; right in red. Right Ear PTA ≈50 dB HL, Left Ear PTA ≈43 dB HL. PTA = Pure Tone Average, dB HL = decibel hearing level.

# **Supplementary Figure 19.-** Predicted clusters in single-cell RNA sequencing controls


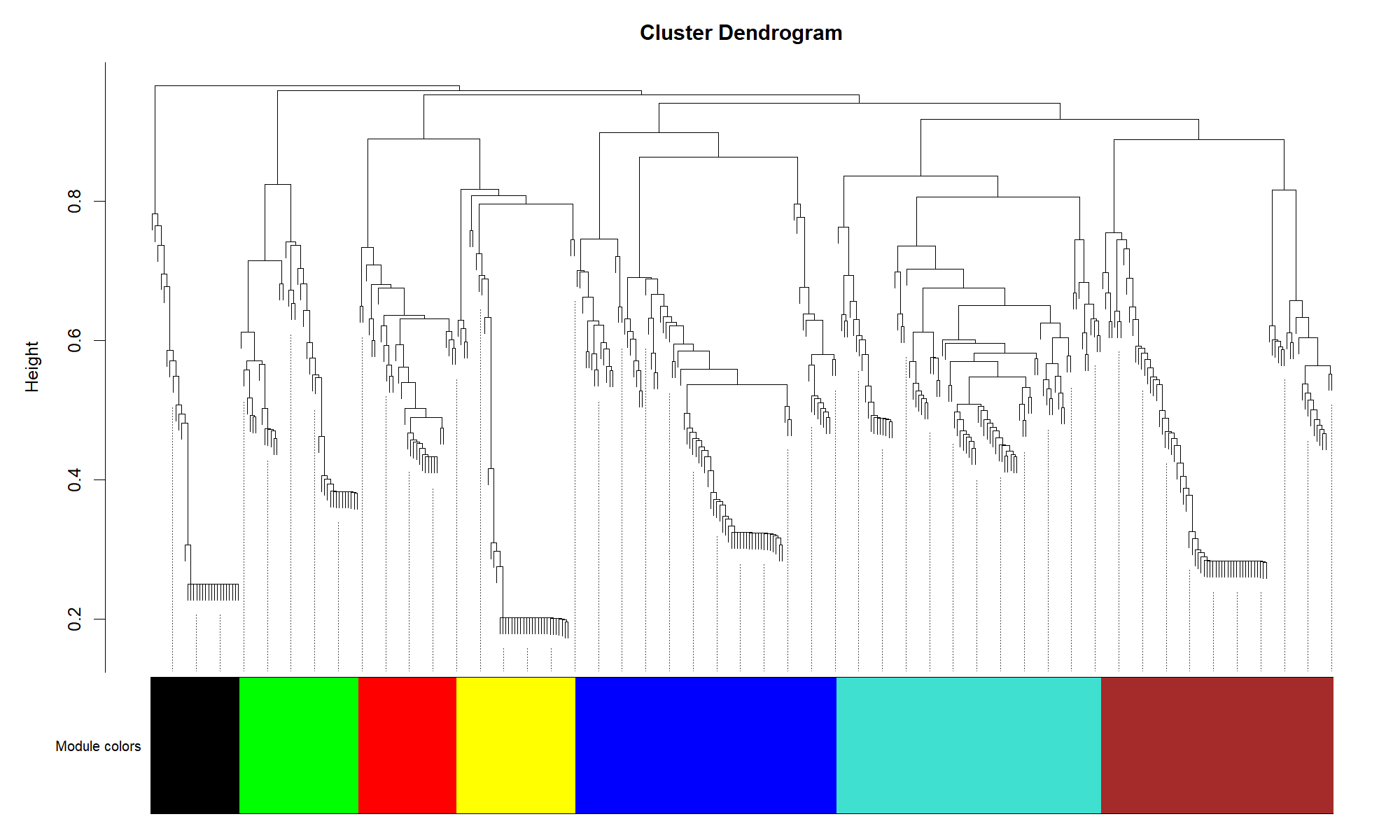


# **Supplementary Figure 20.-** Co-expression of NF-κB pathaway genes in the controls single-cell RNA sequecing predicted cluster


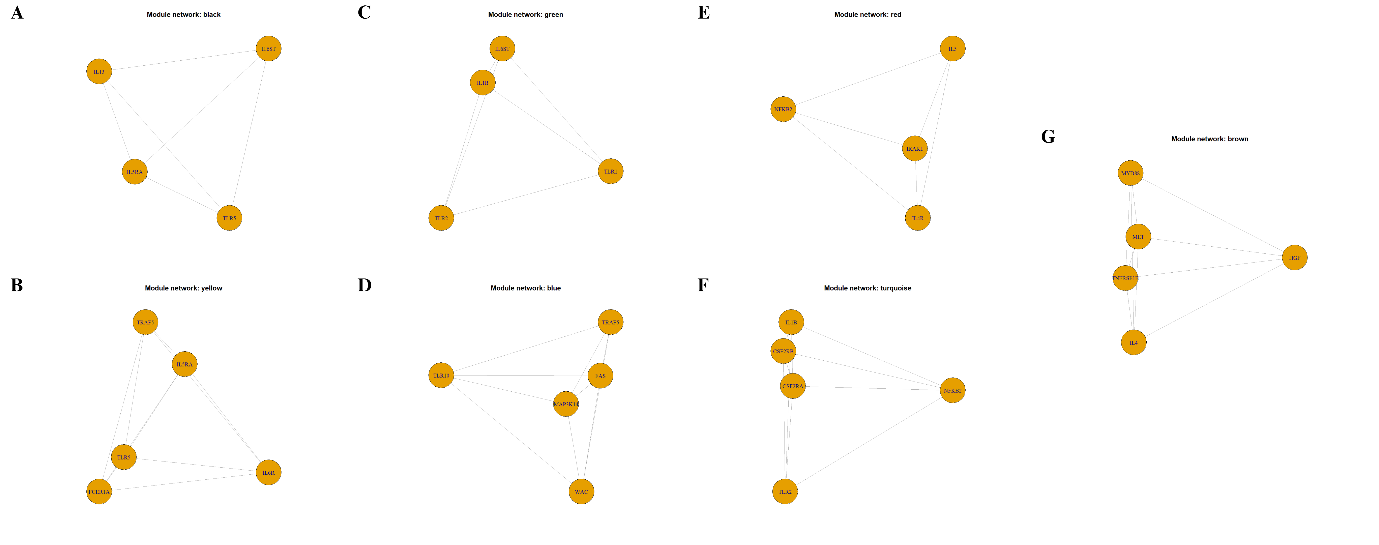


# **Supplementary Figure 21.-** Predicted clusters in single-cell RNA sequencing Menire Disease


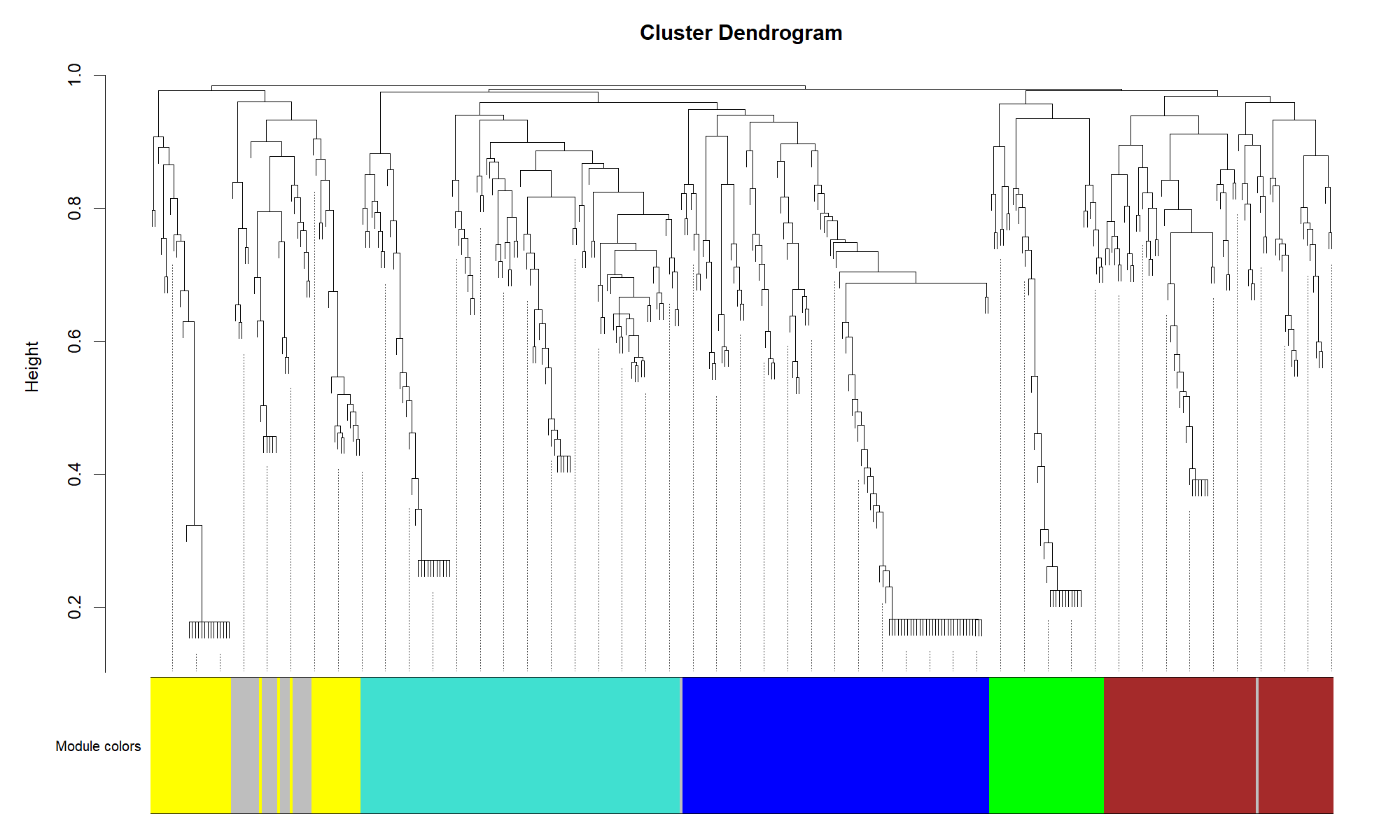


# **Supplementary Figure 22.-** Co-expression of NF-κB pathaway genes in the Menire Disease single-cell RNA sequecing predicted cluster


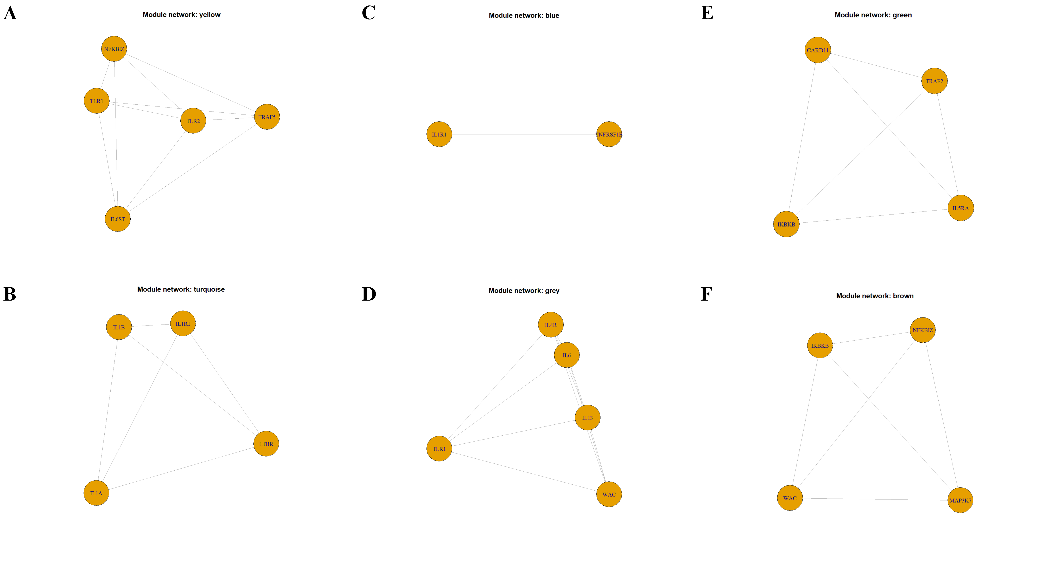


# **Supplementary Figure 23.-** Docked *TLR9*-*TLR9* models. (A) Wild-type – wild-type *TLR9* dimer. (B) Wild-type – mutant *TLR9* dimer. (C) Mutant – mutant *TLR9* dimer.


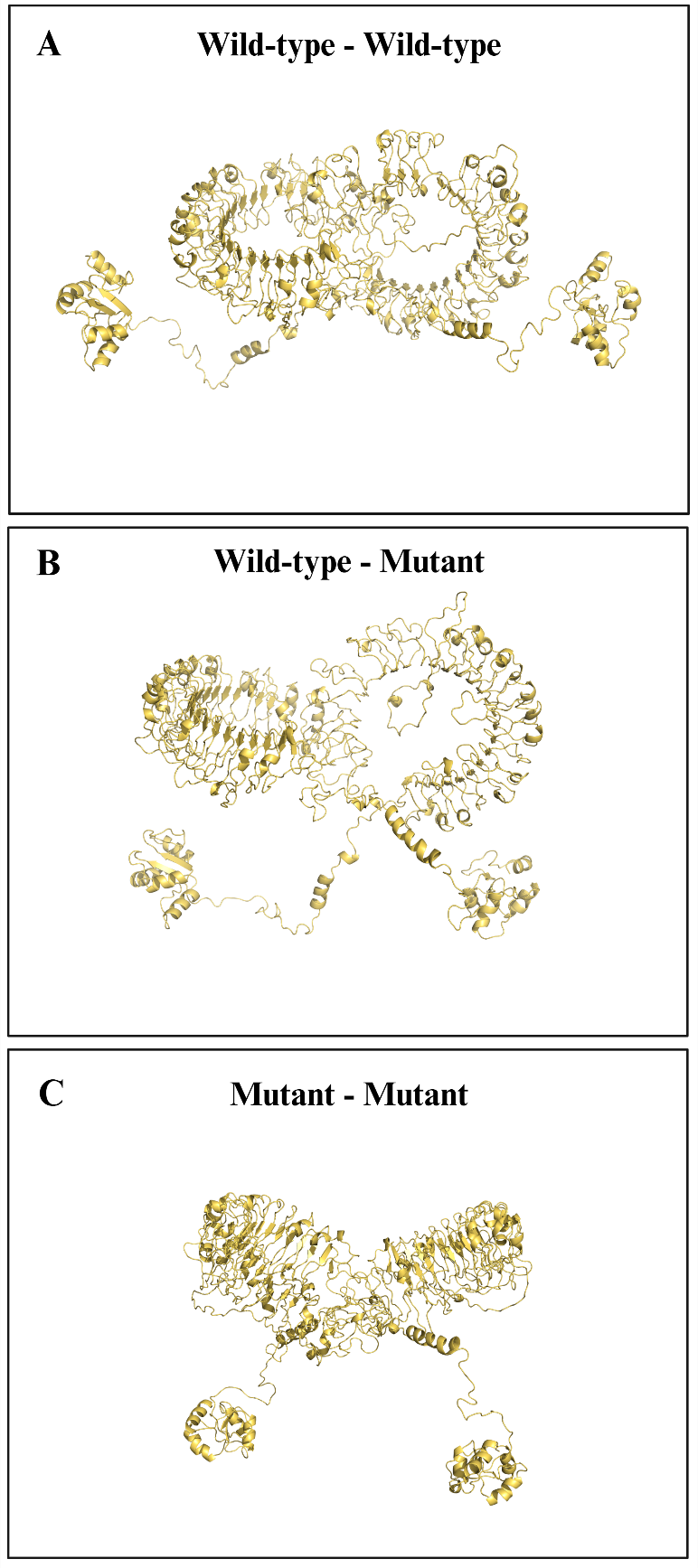


# **Supplementary Figure 24.-** Docked *TNFRSFB*-*TRAF2* models. (A) Wild-type – wild-type *TNFRSFB*-*TRAF2*. (B) Wild-type – mutant *TNFRSFB*-*TRAF2*.


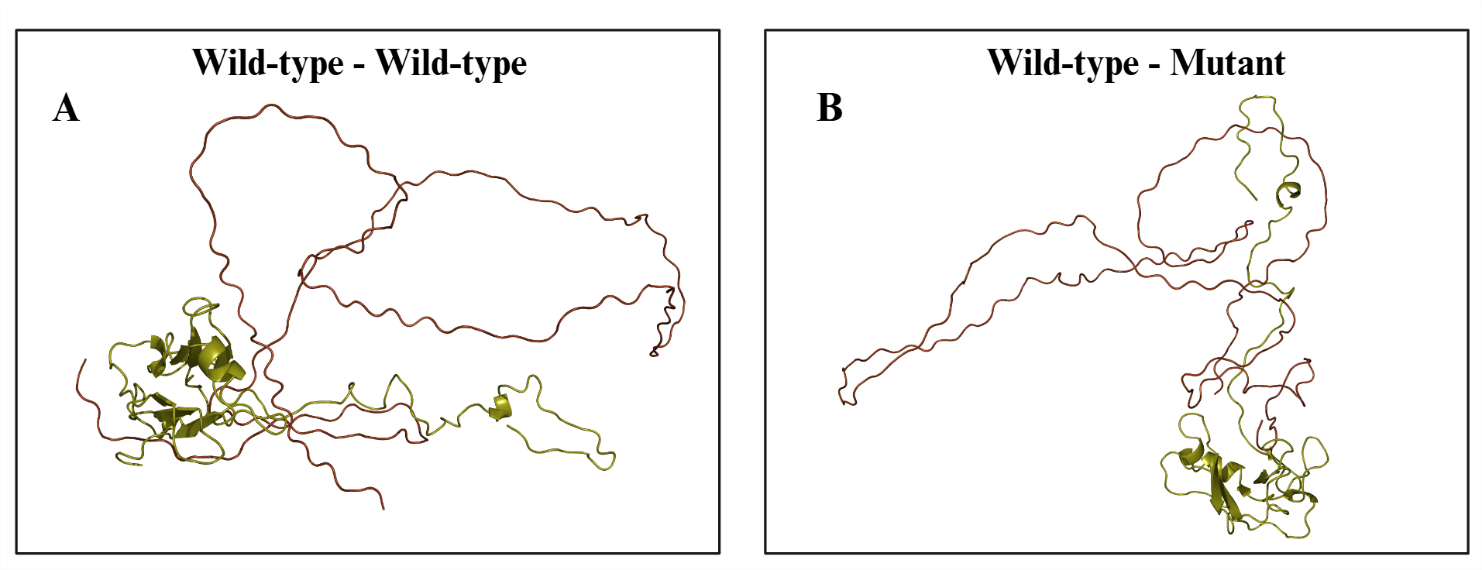

Supplement: Supplementary file 2 [file Table2.docx]
